# Dual Regulation of the Mitotic Exit Network (MEN) by PP2A-Cdc55 Phosphatase

**DOI:** 10.1371/journal.pgen.1003966

**Published:** 2013-12-05

**Authors:** Barbara Baro, Jose-Antonio Rodriguez-Rodriguez, Ines Calabria, María Luisa Hernáez, Concha Gil, Ethel Queralt

**Affiliations:** 1 Cell Cycle Group, Cancer Epigenetics and Biology Program (PEBC), Institut d'Investigacions Biomèdica de Bellvitge (IDIBELL), L'Hospitalet de Llobregat, Barcelona, Spain; 2 Unidad de Proteómica, Parque Científico de Madrid, Facultad de Farmacia, Universidad Complutense de Madrid, Madrid, Spain; The University of North Carolina at Chapel Hill, United States of America

## Abstract

Exit from mitosis in budding yeast is triggered by activation of the key mitotic phosphatase Cdc14. At anaphase onset, the protease separase and Zds1 promote the downregulation of PP2A^Cdc55^ phosphatase, which facilitates Cdk1-dependent phosphorylation of Net1 and provides the first wave of Cdc14 activity. Once Cdk1 activity starts to decline, the mitotic exit network (MEN) is activated to achieve full Cdc14 activation. Here we describe how the PP2A^Cdc55^ phosphatase could act as a functional link between FEAR and MEN due to its action on Bfa1 and Mob1. We demonstrate that PP2A^Cdc55^ regulates MEN activation by facilitating Cdc5- and Cdk1-dependent phosphorylation of Bfa1 and Mob1, respectively. Downregulation of PP2A^Cdc55^ initiates MEN activity up to Cdc15 by Bfa1 inactivation. Surprisingly, the premature Bfa1 inactivation observed does not entail premature MEN activation, since an additional Cdk1-Clb2 inhibitory signal acting towards Dbf2-Mob1 activity restrains MEN activity until anaphase. In conclusion, we propose a clear picture of how PP2A^Cdc55^ functions affect the regulation of various MEN components, contributing to mitotic exit.

## Author Summary

Cell cycle studies over the years have tried to elucidate the molecular mechanisms behind cell division, one of the most highly regulated of all cell processes, which ensures life in all organisms. Protein phosphorylation emerged as a key regulatory mechanism in the cell cycle. The highly conserved family of cyclin-dependent kinases, the Cdks, are considered the main component of the cell cycle control system. However, it has become clear that opposing phosphatases also play a key role in determining the phosphorylation state of the proteins. Cells enter mitosis when mitotic Cdk activity increases, having its pick of activity during metaphase. To exit mitosis, cells must coordinate chromosome segregation with Cdk inactivation processes involving the activation of protein phosphatases. Here we show that the phosphatase PP2A regulates the mitotic exit network (MEN) by counteracting the phosphorylation of Bfa1 and Mob1. Our findings provide new insights into the mechanism by which PP2A-Cdc55 functions affect the regulation of various MEN components that contribute to mitotic exit. The core signalling elements of the MEN, SIN and Hippo pathways are highly conserved. Therefore, studies of MEN regulation will contribute to our understanding of MEN-related pathways in other organisms.

## Introduction

During most of the cell cycle, Cdc14 is kept inactive and sequestered in the nucleolus through binding to its inhibitor Net1 [1], [2]. Two pathways, FEAR (Cdc14 early anaphase release) and MEN (mitotic exit network), activate Cdc14, thereby promoting its release from the nucleolus in early and late anaphase, respectively. Both pathways promote Cdc14 activation by phosphorylating Net1, since the phosphorylated form of Net1 has a low affinity for Cdc14 and loses its ability to inhibit it [3]–[5]. Many proteins, including separase, Cdk1, PP2A^Cdc55^ (type 2A protein phosphatase), Zds1, Slk19, Spo12 and Fob1, have been implicated in early anaphase Cdc14 release (reviewed in [6]–[8]). Several mutants in the FEAR pathway delay the release of Cdc14 from the nucleolus. At early anaphase, upon APC^Cdc20^ (anaphase-promoting complex) activation, securin is degraded by the proteasome and separase is activated, allowing sister chromatid segregation and FEAR-Cdc14 release. The protease separase, the main component of FEAR, allows the Cdk1-dependent phosphorylation of Net1 by downregulating the phosphatase PP2A^Cdc55^ [9]. Zds1 and Zds2 are PP2A-interacting proteins that also participate in the downregulation of PP2A^Cdc55^ [10], [11]. Once Cdk1 activity starts to decline, cells require the MEN pathway to keep Net1 phosphorylated and Cdc14 fully active. The MEN is a GTPase-driven signaling cascade that is associated with the spindle pole body (SPB) [12]–[19]. The main switch of this cascade is the small G protein Tem1 and its regulators: a two-component GAP Bub2–Bfa1, and the putative exchange factor Lte1. Upon activation, Tem1 promotes activation of the Cdc15 protein kinase, which in turn activates the Dbf2–Mob1 kinase complex via phosphorylation [20]. It has recently been found to occur in two steps: Cdc15 first creates phospho-docking sites on the MEN scaffold protein Nud1 and Nud1 phosphorylation recruits Dbf2–Mob1 to SPBs followed by Cdc15-dependent activation of Dbf2–Mob1 [21]. An additional function of the Dbf2–Mob1 complex is to phosphorylate Cdc14 at sites adjacent to its nuclear localization sequence, thereby retaining Cdc14 in the cytoplasm [22]. In an unperturbed cell cycle, the Bub2–Bfa1 complex inhibits the MEN until the Cdc5 Polo kinase inactivates it by phosphorylation. Upon activation of the spindle position checkpoint (SPOC), Bfa1 is phosphorylated by the kinase Kin4 [23]. Kin4 inhibits MEN activation by a phosphorylation that protects Bfa1 from the inhibitory phosphorylation of Cdc5, effectively locking Bub2–Bfa1 in an active state. As a consequence, the MEN pathway is kept inactive until the spindle checkpoint signal is abrogated [24]–[27]. In addition, Cdk1 negatively regulates the function of the MEN components Cdc15 and Mob1 [28], [29]. The first burst of Cdc14 released induced by FEAR, eventually dephosphorylates Cdc15, which further activates Cdc14 [17], [28], [30], [31]. Lte1, in addition to being a putative guanine nucleotide exchange factor for Tem1, participates in the control of Bfa1 localization and cell polarization [32]. Bub2–Bfa1 localizes asymmetrically to the daughter SPB (dSPB) [33], [34] and this asymmetry is required to recruit MEN components to the dSPB during mitosis [35]. Bfa1 phosphorylation during anaphase induces asymmetric localization onto the dSPB [24], [36], [37]. Tem1 is also bound asymmetrically to the dSPB during anaphase and its association with the SPBs is essential for mitotic exit [33], [38], [39]. Moreover, in early anaphase, Cdc15 kinase recruits Cdk1 to the mother spindle pole body (mSPB), while conversely, Cdk1 negatively regulates binding of Cdc15 to the mSPB [29].

Here, we propose that PP2A^Cdc55^ phosphatase acts as a functional link between FEAR and MEN, due to its action on Bfa1 and Mob1. PP2A^Cdc55^ downregulation at anaphase onset facilitates Bfa1 inactivation and thereby initiates the MEN pathway up to the Cdc15 kinase. Premature Bfa1 inactivation observed after PP2A^Cdc55^ inactivation does not entail precocious MEN activation, since the downstream MEN effectors Cdc15 and Dbf2–Mob1 are kept inactive by Cdk1–Clb2 phosphorylation. In this way, Cdk1–Clb2 restrains MEN activity until mid-late anaphase allowing a consecutive order of FEAR- and MEN-Cdc14 functions.

## Results

### PP2A^Cdc55^ counteracts Bfa1 phosphorylation

The Bub2–Bfa1 complex keeps MEN inactive by inhibiting Tem1. Phosphorylation of Bfa1 by Polo kinase, Cdc5, contributes to MEN activation in an anaphase-specific manner [24], [30]. However, there is no evidence of upregulation of Polo activity during anaphase, and preliminary results suggested that downregulation of PP2A^Cdc55^ could facilitate anaphase-specific phosphorylation of Bfa1 [9].

**Figure 1:**
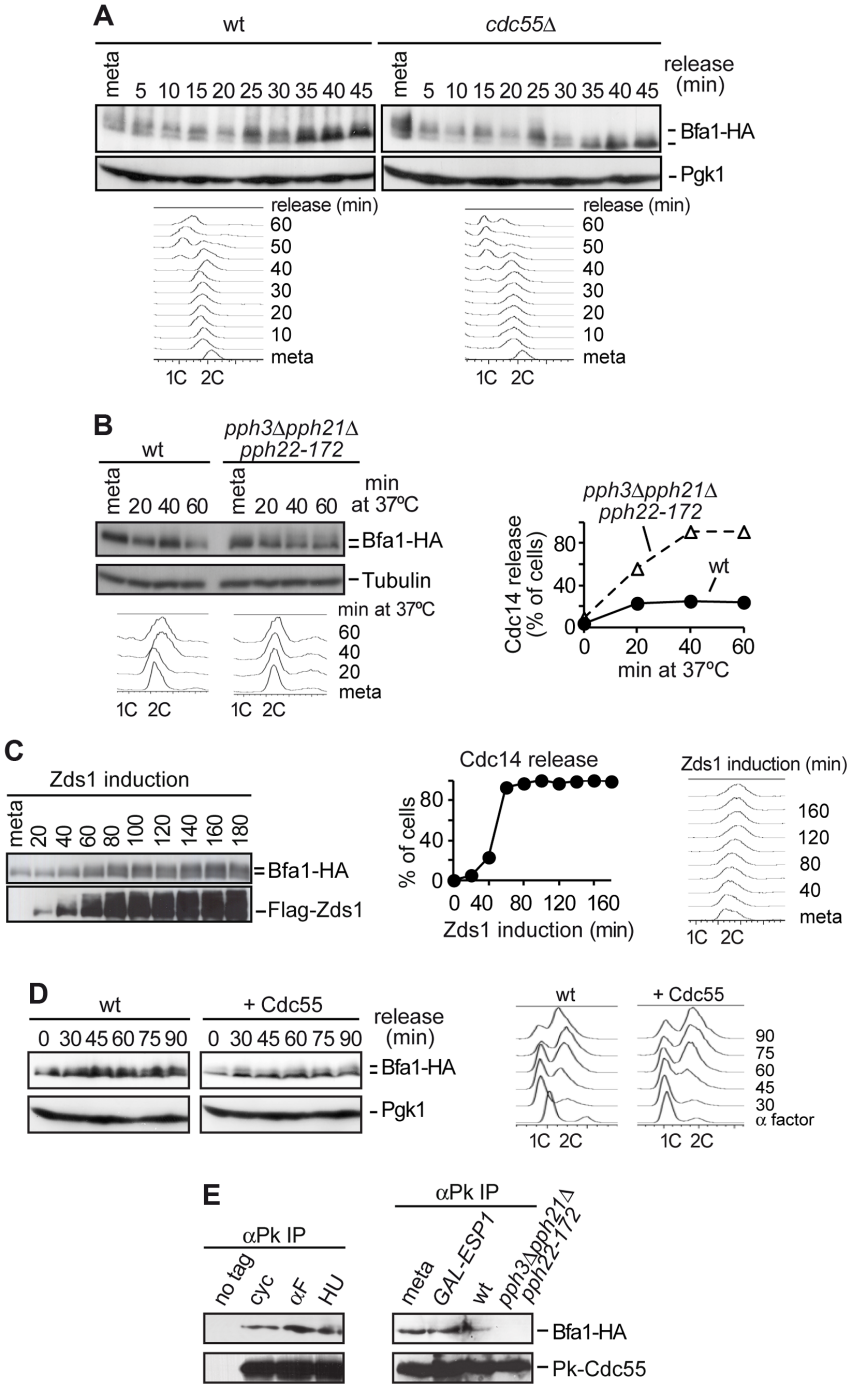
PP2A^Cdc55^ counteracts Bfa1 phosphorylation. (A) Premature Bfa1 phosphorylation in metaphase in the absence of PP2A^Cdc55^. Strains Y513 (*MATa MET-CDC20 CDC14-Pk_9_ BFA1-HA_6_*) and Y514 (as Y513, but *cdc55*Δ) were arrested in metaphase by Cdc20 depletion and released into synchronous anaphase by Cdc20 reintroduction. Bfa1 phosphorylation was analyzed by western blot. Pgk1 served as loading control. FACS profiles were used as a control of anaphase progression. At least 100 cells were scored at each time. Tubulin served as a loading control. (B) PP2A^Cdc55^ catalytic activity is required to keep Bfa1 under-phosphorylated in metaphase. Strains Y1021 (*MATa Pk_3_-CDC55 BFA1-HA_6_*) and Y1020 (*MATa pph3*Δ *pph21*Δ *pph22-172 BFA1-HA_6_*) were arrested in metaphase by nocodazole treatment and shifted to 37°C. (C) Zds1-dependent inactivation of PP2A^Cdc55^ promotes Bfa1 phosphorylation. Strain Y597 (*MATa MET-CDC20 GAL1-Flag_3_-ZDS1 CDC14-Pk_9_ BFA1-HA_6_*) was arrested in metaphase by Cdc20 depletion and Zds1 ectopic expression was induced by addition of galactose. Bfa1 phosphorylation and Zds1 expression levels were analyzed by western blot. (D) Overexpression of Cdc55 restrains Bfa1 phosphorylation. Strain Y1190 (*MATa* 4×*GAL1-CDC55 CDC14-Pk_9_ BFA1-HA_6_*) was arrested at G1 with α-factor and released into a synchronous cell cycle. Half of the culture was released into medium containing galactose to induce Cdc55 overexpression and the other half was released without galactose as a control. Bfa1 phosphorylation was analyzed by western blot and Pgk1 served as a loading control. FACS profiles were used as a control of cell cycle progression. (E) Cdc55 and Bfa1 interact. Co-immunoprecipitation of Cdc55 and Bfa1 was analyzed in protein extracts from strains Y1021 (*MATa Pk_3_-CDC55 BFA1-HA_6_*), Y1146 (*MATa pph3*Δ *pph21*Δ *pph22-172 Pk_3_-CDC55 BFA1-HA_6_*) and Y1145 (*MATa MET-CDC20 GAL1-Flag_3_-ESP1 Pk_3_-CDC55 BFA1-HA_6_*). Strains Y1146 and Y1021 were treated at 37°C for 120 min. Strain Y1145 was arrested in metaphase by Cdc20 depletion, and separase ectopic expression was induced for 120 min. Protein extracts from strain Y1063 (*MATα BFA1-HA_6_*) lacking a Pk epitope on Cdc55 served as a control.

To investigate the role of PP2A^Cdc55^ in regulating MEN activity, we studied the dephosphorylation of Bfa1 by PP2A^Cdc55^. First, we confirmed that Bfa1 was already phosphorylated in metaphase in a *cdc55*Δ mutant (Figure 1A and [9]). Wild-type and *cdc55*Δ cells were synchronized at the metaphase-to-anaphase transition by Cdc20 depletion. Bfa1 electrophoretic mobility and Cdc14 release were analyzed after releasing cells into anaphase. In wild-type cells, the electrophoretic mobility of Bfa1 decreases during anaphase. Bfa1 becomes dephosphorylated upon exit from mitosis. In contrast, Bfa1 already has a slower migration form in metaphase in *cdc55*Δ cells. This result indicates that Bfa1 already has an anaphase-like phosphorylation pattern in metaphase, suggesting that PP2A^Cdc55^ prevents Bfa1 phosphorylation.

Secondly, we studied Bfa1 phosphorylation in the absence of PP2A^Cdc55^ phosphatase catalytic activity. We used a strain carrying a deletion of *PPH21*, a temperature-sensitive *pph22-172* allele that inactivates the two PP2A^Cdc55^ catalytic subunits, and a deletion of *PPH3* (since Pph3 phosphatase can partly compensate for PP2A^Cdc55^ activity in budding yeast [40]). Cells were arrested in metaphase with nocodazole and shifted to the restrictive temperature in order to inactivate PP2A^Cdc55^. Bfa1 phosphorylation increased after shifting to the restrictive temperature in *pph3*Δ*pph21*Δ*pph22-172* cells (Figure 1B). Upon treatment at 37°C, Cdc14 was released from the nucleolus, confirming PP2A^Cdc55^ inactivation. This result suggests that PP2A^Cdc55^ catalytic activity is required to keep Bfa1 underphosphorylated in metaphase. However, in this strain the activity of the PP2A complex containing the Rts1 regulatory subunit is also impaired. Therefore, we cannot rule out a contribution of the PP2A^Rts1^ to the Bfa1 phosphorylation in this specific *pph3*Δ*pph21*Δ*pph22-172* mutant. However, Bfa1 phosphorylation is not affected in an *rts1*Δ mutant [41], indicating that PP2A^Rts1^ complexes do not counteract Bfa1 phosphorylation.

Thirdly, we determined Bfa1 phosphorylation status under Zds1 overexpression, which promotes PP2A^Cdc55^ inactivation [10]. Cells were arrested in metaphase by Cdc20 depletion, and Zds1 expression was induced by galactose addition. Elongation of anaphase spindles was not observed as a control of the cells being blocked in metaphase during the experiment. Bfa1 became phosphorylated 40–60 minutes post-induction when Cdc14 was released, representing a marker of PP2A^Cdc55^ phosphatase inactivation (Figure 1C). This indicates that Zds1-dependent inactivation of PP2A^Cdc55^ promotes Bfa1 phosphorylation. Similar results were obtained when we induced inactivation of PP2A^Cdc55^ by separase ectopic expression (Figure S1). Next, we examined whether forcing Cdc55 expression would specifically remove Cdc5-induced phosphorylation of Bfa1. Cells containing *CDC55* under the *GAL1* promoter were arrested in G1 with α-factor and released from the G1 block in the presence of galactose (Figure 1D). Bfa1 became phosphorylated 45 min after the G1 release in the half of the culture that was kept without galactose, as a control. Conversely, Bfa1 was not phosphorylated in cells overexpressing Cdc55. These results indicate that PP2A^Cdc55^ phosphatase counteracts Bfa1 phosphorylation. Finally, to determine whether Bfa1 is a PP2A^Cdc55^ substrate, we examined whether Cdc55 and Bfa1 physically interact. Co-immunoprecipitation experiments showed that Bfa1 co-purified with Cdc55 at all stages of the cell cycle (Figure 1E). Moreover, the Cdc55 and Bfa1 interaction was impaired in the absence of PP2A catalytic activity (*pph3Δpph21Δpph22-172* mutant at restrictive temperature) due to the loss of integrity of the PP2A trimeric complex in this mutant. Taken together, these results suggest that Bfa1 is likely to be an *in vivo* substrate of PP2A^Cdc55^.

Bfa1 phosphorylation in the normal cell cycle depends on Cdc5 Polo kinase activity, so we expected that the premature phosphorylation of Bfa1 observed upon PP2A^Cdc55^ inactivation would depend on Cdc5. To test this, we compared Bfa1 electrophoretic mobility after Zds1 induction in wild-type and *cdc5-14* mutant cells (Figure S2). Cells were arrested in metaphase by Cdc20 depletion, and shifted to the restrictive temperature. Galactose was then added to induce Zds1 ectopic expression. Bfa1 was already phosphorylated in metaphase in both strains due to the prolonged incubation at 37°C. This temperature-dependent Bfa1 phosphorylation was also observed in the experiment illustrated in Figure 1B in which the cells were incubated at 37°C for at least 120 min. Bfa1 hyperphosphorylation was clearly observed upon Zds1 induction in wild-type cells, but was not induced in *cdc5-14* mutant cells during the induction time-course. This result suggests that Bfa1 phosphorylation after PP2A^Cdc55^ inactivation depends on Cdc5 Polo kinase. To determine whether PP2A^Cdc55^ also contributes to Bfa1 phosphorylation upon spindle position checkpoint activation, we compared Bfa1 electrophoretic mobility in wild-type and *kin4*Δ cells after Zds1 induction. Both strains showed Bfa1 hyperphosphorylation as Zds1 accumulated (Figure S3). This result indicates that the Bfa1 phosphorylation observed upon PP2A^Cdc55^ inactivation does not depend on kinase Kin4. We can conclude that PP2A^Cdc55^ counteracts Cdc5-dependent Bfa1 phosphorylation.

### PP2A^Cdc55^ regulates Mob1 phosphorylation

**Figure 2:**
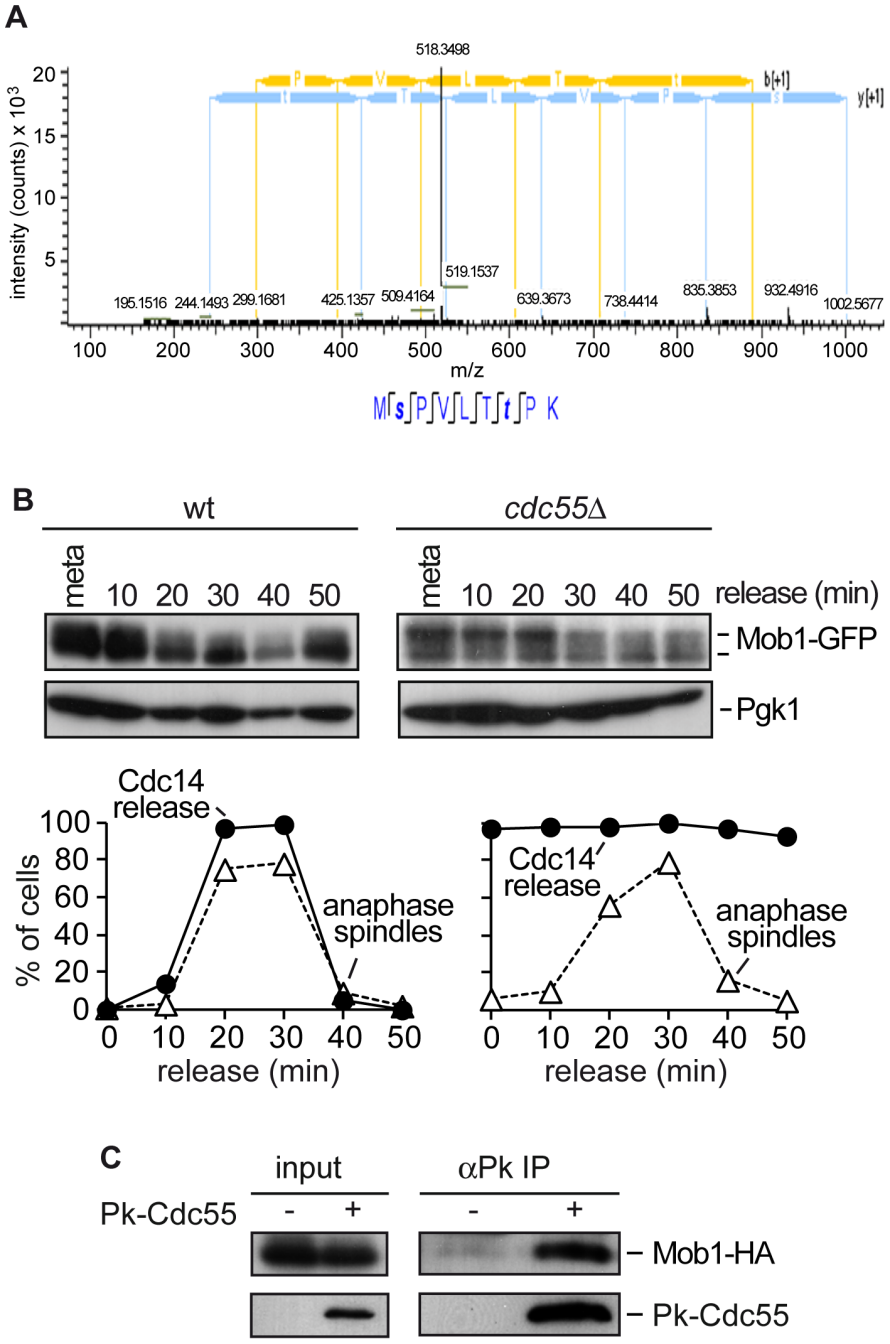
PP2A^Cdc55^ regulates Mob1 phosphorylation. (A) MS^2^ spectrum of peptide MpSPVLTpTPK, which includes the dually phosphorylated Ser and Thr residues. Peptide sequence and assigned fragment ions *y* (in blue) and *b* (in yellow) are indicated (pRS site probabilities = 98.8%; determined by Proteome Discoverer Software v 1.3). (B) Cdc55 counteracts Mob1 phosphorylation. Strains Y1109 (*MATa MET-CDC20 CDC14-Pk_9_ eGFP*-*MOB1*) and Y1110 (as Y1109, but *cdc55*Δ) were released into a synchronous anaphase by Cdc20 depletion and reintroduction. Mob1 phosphorylation was analyzed by western blot. (C) Cdc55 and Mob1 interact. Co-immunoprecipitation of Cdc55 and Mob1 was analyzed in protein extracts from Y1106 (*MATa Pk_3_-CDC55 MOB1-HA_6_*). Protein extracts from strain Y1104 (*MATa MOB1-HA_6_*) lacking a Pk epitope on Cdc55 served as a control.

To screen for new PP2A^Cdc55^ substrates during mitosis, we performed a global study of the PP2A^Cdc55^ phosphoproteome by a quantitative phosphoproteomic analysis based on SILAC labeling. Wild-type cells were labeled using ^13^C_6_-lysine and ^13^C_6_-arginine (heavy), and *cdc55*Δ mutant cells were grown in the presence of unmodified arginine and lysine (light). Cells were arrested in metaphase and protein extracts were prepared. Phosphopeptides were enriched by SIMAC-based enrichment. Phosphopeptide analysis of the heavy/light-labelled cells was done by LC-MS/MS. An already known PP2A^Cdc55^ substrate Net1 was identified as being hyperphosphorylated in the *cdc55*Δ mutant, suggesting that the technique worked properly. The screening revealed that a phosphopeptide corresponding to Mob1 protein was hyperphosphorylated in *cdc55*Δ mutant cells. The Mob1 peptide contained two S/TP sites: S80 and T85 (Figure 2A). Both sites were detected with the highest confidence (pRS site probability around 100% and q = 0.000168). The T85 is one of the full Cdk1 consensus sites (S/T-P-x-K/R) previously reported to be phosphorylated by Cdk1 [29]. This result suggests that Mob1 could be a PP2A^Cdc55^ substrate. To examine this further we studied the Mob1 phosphorylation levels in a synchronous anaphase in *cdc55Δ* mutant cells. Wild-type and *cdc55*Δ cells were synchronized at the metaphase-to-anaphase transition by Cdc20 depletion. In wild-type cells, Mob1 is dephosphorylated during anaphase, coincident with spindle elongation and Cdc14 release from the nucleolus. In contrast, Mob1 was hyperphosphorylated in metaphase in *cdc55Δ* cells and its high electrophoretic mobility forms were still detected during anaphase (Figure 2B). This result indicates that Mob1 is hyperphosphorylated in metaphase and is not correctly dephosphorylated during anaphase in the absence of PP2A^Cdc55^ activity (despite Cdc14 being already released in metaphase), suggesting that PP2A^Cdc55^ is required to dephosphorylate Mob1 properly. Finally, we examined whether Cdc55 and Mob1 physically interact. Co-immunoprecipitation experiments showed that Mob1 co-purified with Cdc55 (Figure 2C). Together, these results suggest that Mob1 is probably an *in vivo* substrate of PP2A^Cdc55^.

### PP2A^Cdc55^ function is required for proper MEN activation and efficient mitotic exit

**Figure 3:**
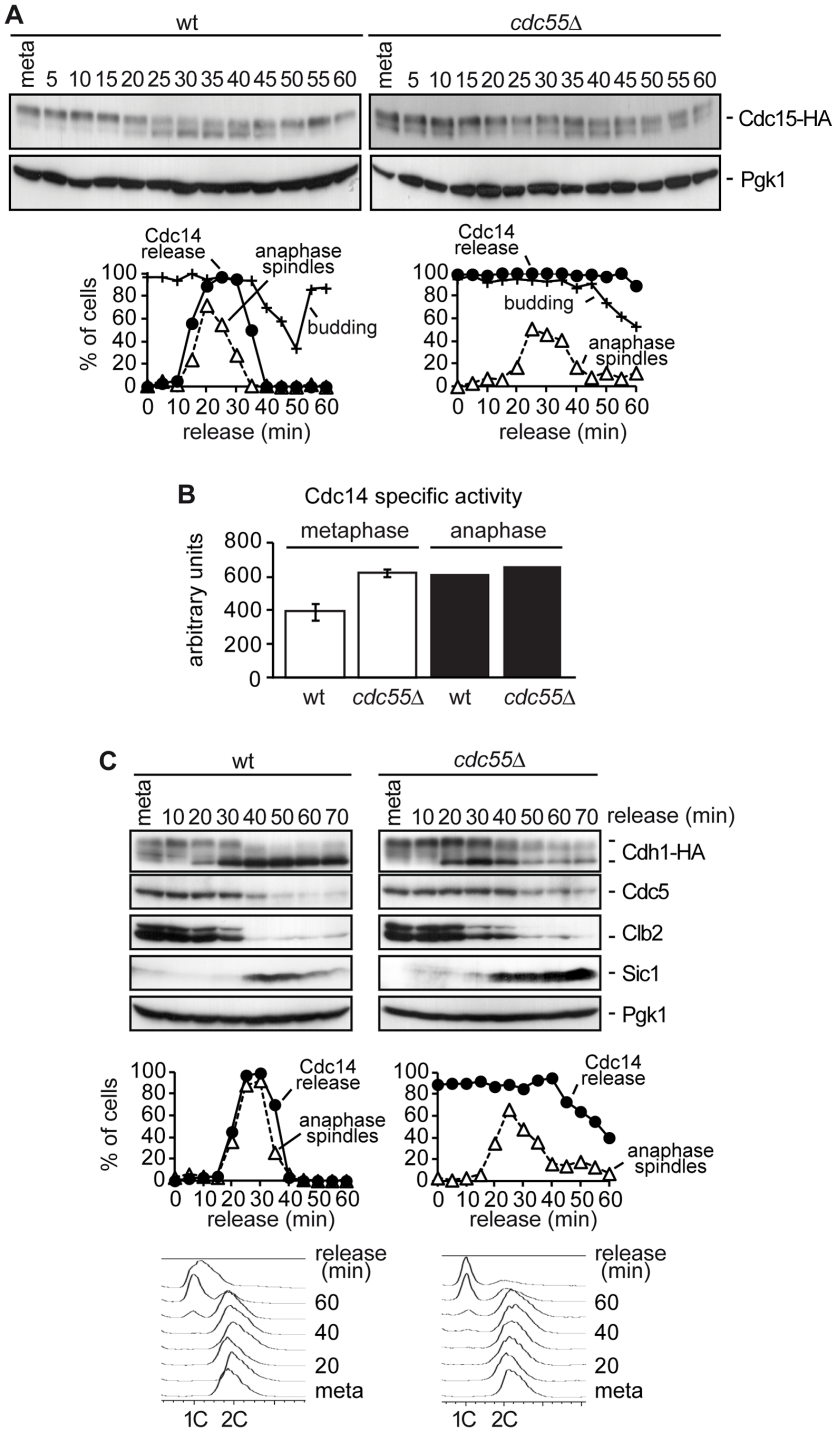
Premature Bfa1 inactivation does not provoke premature exit from mitosis. (A) Cdc15 phosphorylation in the absence of Cdc55. Strains Y2961 (*MATa MET-CDC20 CDC14-Pk_9_ CDC15-HA_6_*) and Y528 (as Y2961, but *cdc55*Δ) were released into a synchronous anaphase by Cdc20 depletion and reintroduction. Cdc15 phosphorylation was analyzed by western blot. The budding index was used to monitor cell cycle progression. Pgk1 served as a loading control. (B) Cdc14 activity in *cdc55*Δ mutant cells. Strains Y2810 (*MATa MET-CDC20 CDC14-*Pk_9_) and Y2118 (as Y2810, but *cdc55*Δ) were arrested in metaphase by Cdc20 depletion. Anaphase samples were taken 20 min after release when more than 80% of the cells showed anaphase spindles. Phosphatase activity from immunopurified Cdc14 was measured as described in the Materials and Methods. Means and standard deviations are shown. (C) The MEN is not prematurely active in the absence of Cdc55. Strains Y547 (*MATa MET-CDC20 CDC14-Pk_9_ HA_3_-CDH1*) and Y548 (as Y547, but *cdc55*Δ) were released into synchronous anaphase by Cdc20 depletion and reintroduction. Cdh1 phosphorylation and Cdc5, Clb2 and Sic1 proteins levels were analyzed by western blot.

The Bub2–Bfa1 complex inhibits the MEN pathway until Cdc5-dependent phosphorylation of Bfa1 alleviates Tem1 inhibition by Bub2–Bfa1 [24]. Therefore, we would expect the premature phosphorylation of Bfa1 observed in the absence of PP2A^Cdc55^ activity to cause premature MEN activation. If so, activation of the MEN cascade would happen earlier in the *cdc55*Δ mutant than in the wild-type. However, on the basis of anaphase FACS dynamics (Figure 1A), we found no evidence of accelerated mitotic exit in *cdc55*Δ mutant cells compared with wild-type cells. Cdc15 is dephosphorylated during exit from mitosis by Cdc14 [14]–[17], [28]. The Cdc15 dephosphorylation event has often been used as a MEN activation marker, so if MEN is prematurely activated in the absence of PP2A^Cdc55^ activity, we should observe premature dephosphorylation of Cdc15. Wild-type and *cdc55*Δ mutant cells were synchronized at the metaphase-to-anaphase transition by Cdc20 depletion, and Cdc15 phosphorylation was analyzed by western blot. In wild-type cells, the electrophoretic mobility of Cdc15 increased, being especially evident between 20 and 40 minutes, when the cells were in anaphase (Figure 3A). At these times, the MEN was active (as indicated by the anaphase spindle dynamics) and Cdc15 was dephosphorylated, as previously reported. Interestingly, in the *cdc55*Δ mutant, we detected a faster migration form of Cdc15 already at metaphase. Note that this could be reminiscent of Cdc14 being released from the nucleolus already in metaphase in the *cdc55*Δ mutant. However, Cdc15 dephosphorylation was never as efficient as the wild-type cells in anaphase. We conclude that, in the absence of PP2A^Cdc55^ activity, Cdc15 is prematurely dephosphorylated, although MEN is not prematurely activated, since mitotic exit is not accelerated.

We did not observe any further increase in Cdc15 mobility during anaphase. One explanation could be that the Cdc14 prematurely released from the nucleolus in metaphase in the *cdc55*Δ mutant was not active as phosphatase, and therefore, PP2A^Cdc55^ could be required for full activation of Cdc14 during anaphase. To resolve this matter we measured Cdc14 activity *in vitro* in wild-type and *cdc55*Δ mutant strains in metaphase and anaphase cells. As can be observed in Figure 3B, Cdc14 activity in the *cdc55*Δ mutant is equal in metaphase and anaphase cells. More interestingly, the Cdc14 activity in the *cdc55*Δ mutant is equivalent to anaphase-Cdc14 in wild-type cells. Therefore, this result indicates that Cdc14 activity is not impaired in *cdc55*Δ mutant cells; indeed, Cdc14 activity has similar levels to the fully active Cdc14 in anaphase. However, although Cdc14 is active in the *cdc55*Δ mutant, the susceptibility of the Cdc14 substrates to dephosphorylation also depends on kinase levels. The Cdk1/Cdc14 ratio over the course of mitotic exit is read out by Cdk substrates that respond by dephosphorylation at distinct thresholds [42].

Exit from mitosis and cytokinesis require full activation of Cdc14 by the MEN pathway. Cdc14 promotes the inactivation of the Cdk1–Clb2 complex at the end of mitosis by dephosphorylating Cdh1 an activator of the APC complex in late anaphase. Cdh1 is responsible for the degradation of mitotic B-type cyclins, Cdc5, Swi5, Sic1 and Cdc20, among numerous other proteins, including several involved in spindle stability and assembly [43]–[49].

To further characterize how PP2A^Cdc55^ phosphatase activity impinges on MEN activation, we next studied late anaphase events that require MEN activation. Wild-type and *cdc55*Δ cells were arrested in metaphase by Cdc20 depletion and then released into synchronous anaphase by Cdc20 reintroduction. Cdh1 dephosphorylation, Cdc5 and Clb2 degradation, and Sic1 accumulation were analyzed as markers of late anaphase events by western blot. Wild-type cells showed a decrease of the slower migration forms of Cdh1 at 20–40 minutes when cells were in anaphase (Figure 3C), indicating that Cdh1 becomes dephosphorylated during this period. By contrast, in *cdc55*Δ mutant cells, phosphorylated forms of Cdh1 persisted during anaphase and only a partial dephosphorylation of Cdh1 was observed. This suggests that Cdh1, a direct target of MEN, is not correctly activated in the absence of PP2A^Cdc55^ activity.

To confirm this result, we also studied the activity of APC^Cdh1^ with respect to its substrates, Cdc5 and Clb2. In wild-type cells, Cdc5 and Clb2 degradation started when Cdh1 became active in anaphase (at 30–40 minutes). However, Cdc5 protein levels were almost constant in the *cdc55*Δ mutant cells in anaphase. On the other hand, Clb2 was only partially degraded at 30 minutes in the *cdc55*Δ mutant cells, indicative of the first Clb2 degradation by APC^Cdc20^. We conclude that APC^Cdh1^ does not degrade Cdc5 and Clb2 efficiently in cdc55Δ mutant cells.

Finally, we studied the accumulation of Sic1 protein levels, the Cdk1 inhibitor. In wild-type cells, Sic1 protein became detectable during anaphase and persisted until the next G1/S phase. Nevertheless, Sic1 was timely accumulated in the *cdc55*Δ mutant cells, but remained constant at later times since *cdc55*Δ mutant cells had a longer G1 phase (see FACS profiles in Figure 1A and [9]). This is consistent with the cells not being able to enter the next S phase until the APC^Cdh1^ substrates have been correctly degraded during G1. Despite the slower kinetics in Cdh1 dephosphorylation and Cdc5 and Clb2 degradation, Sic1 accumulation was probably sufficient to inhibit Cdk1 and to exit from mitosis in the absence of Cdc55, but not to complete mitosis as efficiently as in the wild-type cells. In fact, Cdc14 resequestration was also delayed in *cdc55*Δ mutant cells. Taking these results together, we can conclude that PP2A^Cdc55^ activity is required for efficient MEN activation and mitotic exit.

### PP2A^Cdc55^ downregulation initiates the MEN pathway by facilitating Bfa1 inactivation

In the normal cell cycle, the Bub2–Bfa1 complex localizes asymmetrically at the dSPB in anaphase when Cdc5 Polo kinase phosphorylates Bfa1 and alleviates MEN inactivation [24], [33], [34], [50]. Bfa1 is a phosphoprotein and its asymmetric localization on the dSPB is induced upon Bfa1 phosphorylation [24], [36], [37], [50].

**Figure 4:**
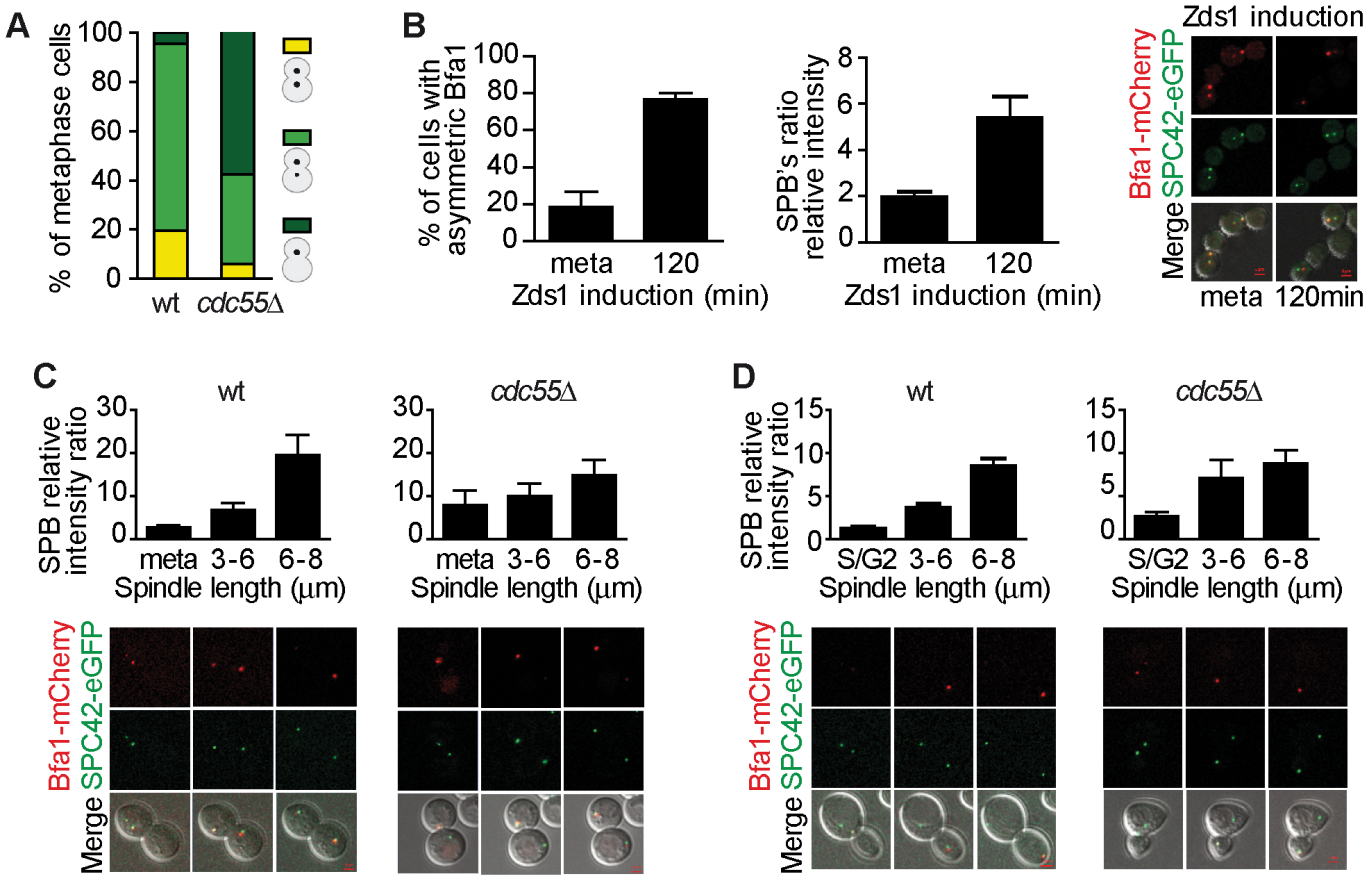
Bfa1 localizes asymmetrically at the dSPB in the absence of PP2A^Cdc55^ activity. (A) Cdc55 deletion causes premature Bfa1 asymmetric localization at the dSPB in metaphase-arrested cells. Strains Y559 (*MATa MET-CDC20 CDC14-Pk_9_ BFA1-eGFP*) and Y560 (as Y559, but *cdc55*Δ) were arrested in metaphase by Cdc20 depletion. Percentages of equal, high/low and single distribution of Bfa1-eGFP were determined. At least 100 cells were scored for each strain. (B) Bfa1 localization becomes asymmetric upon Zds1-dependent inactivation of PP2A^Cdc55^. Strain Y875 (*MATα MET-CDC20 GAL1-Flag_3_-ZDS1 CDC14-Pk_9_ BFA1-mCherry SPC42-YFP*) was arrested in metaphase by Cdc20 depletion and galactose was added to induce Zds1 overexpression. Asymmetric Bfa1-mCherry signal was quantified as the SPB relative intensity ratio as described in the Materials and Methods. (C) Premature Bfa1 asymmetric localization in the absence of Cdc55 in a metaphase-to-anaphase transition. Strains Y951 (*MATa MET-CDC20 CDC28F19 CDC14-myc_9_ BFA1-mCherry SPC42-YFP*) and Y1017 (as Y951, but *cdc55*Δ) were arrested in metaphase by Cdc20 depletion and released into synchronous anaphase by Cdc20 reintroduction. Time-lapse microscopy was used to visualize Bfa1-mCherry localization dynamics. Bfa1 SPB ratios were measured during mitosis progression (n = 15 for WT; n = 10 for *cdc55*Δ). The distance between the two SPBs was used to calculate the spindle length. (D) Premature Bfa1 asymmetric localization in the absence of Cdc55 in a synchronous cell cycle after G1 release. Strains Y876 (*MATa CDC28F19 CDC14-myc_9_ BFA1-mCherry SPC42-YFP*) and Y1006 (as Y876, but *cdc55*Δ) were arrested at G1 with α-factor and released into a synchronous cell cycle. Time-lapse microscopy was performed as in (C) (n = 26 for WT; n = 25 for *cdc55*Δ). Scale bar, 2 µm.

Our results described above indicate that Bfa1 is hyperphosphorylated in the absence of PP2A^Cdc55^ activity, so we next investigated whether Bfa1 also undergoes premature asymmetric localization on the dSPB upon PP2A^Cdc55^ inactivation. First, we examined the Bfa1 localization in wild-type and *cdc55*Δ metaphase-arrested cells containing Bfa1-eGFP (Figure 4A). In wild-type cells, Bfa1 was preferentially symmetrically located on both SPBs in metaphase-arrested cells. Conversely, in the *cdc55*Δ mutant Bfa1 was preferentially located on the dSPB in 58% of the cells. Therefore, Bfa1 was mainly asymmetrically localized on the dSPB in the absence of Cdc55.

Next, we induced Bfa1 phosphorylation by Zds1-dependent inactivation of PP2A^Cdc55^ in cells containing Bfa1-mCherry and Spc42-YFP as an SPB marker. Upon Zds1 ectopic expression, Bfa1 localization was asymmetric in 73% of the cells (Figure 4B and Figure S4). To assess the extent of Bfa1 asymmetric localization we quantified the signal intensity ratio between the two SPBs (dSPB/mSPB relative intensity; hereafter the SPB ratio). The SPB ratio reached a value of 5.2 120 min after Zds1 induction. Our results indicate that Bfa1 becomes prematurely asymmetrically localized in the absence of PP2A^Cdc55^ function.

Lastly, we studied the changes in Bfa1 localization during the cell cycle in the absence of PP2A^Cdc55^ activity. As a first approach, we synchronized cells at the metaphase-to-anaphase transition by Cdc20 depletion (Figure 4C). In wild-type cells, Bfa1 became asymmetric in anaphase when the cells had a spindle length >6 µm and a mean SPB ratio of 20. Conversely, in *cdc55*Δ mutant cells the SPB ratios were already >7 in metaphase. Next, we analyzed Bfa1 localization in cell-cycle progression after synchronous release from G1 in wild-type and *cdc55*Δ mutant cells bearing the *CDC28^Y19F^* allele that is refractory to Cdk1 inhibition (Figure 4D). *cdc55*Δ cells show a delay in progression through mitosis because their Cdk1 activity is compromised by inhibitory Cdc28–Y19 phosphorylation [51]. However, the *cdc55*Δ *CDC28^Y19F^* mutant shows the premature Cdc14 release from the nucleolus in metaphase that is typical of *cdc55*Δ deletion mutants [9]. In wild-type cells, the Spc42-YFP signal split around S phase, indicative of the SPB duplication, but the Bfa1-mCherry signal could not be detected (Figure 4D, S/G2 column). Bfa1-mCherry was subsequently visualized transiently in both SPBs. When the cells had a spindle length >6 µm, Bfa1-mCherry was detected only in the dSPB, consistent with other published results [50]. By contrast, in *cdc55*Δ *CDC28^Y19F^* mutant cells, it was detected on the SPBs shortly after the Spc42-YFP signal had been duplicated. As soon as the Bfa1-mCherry signal was visualized, Bfa1 was asymmetrically loaded onto the dSPB (SPBs ratios of approximately 3). When the cells reached mitosis, the Bfa1-mCherry signal increased and the asymmetry became more evident. These results further confirmed the premature asymmetric Bfa1 localization in the absence of PP2A^Cdc55^ function. Thus, PP2A^Cdc55^ downregulation initiates MEN signalling by allowing Cdc5-dependent phosphorylation of Bfa1 and its asymmetric localization.

### MEN signalling is transduced up to Cdc15 kinase after PP2A^Cdc55^ downregulation

**Figure 5:**
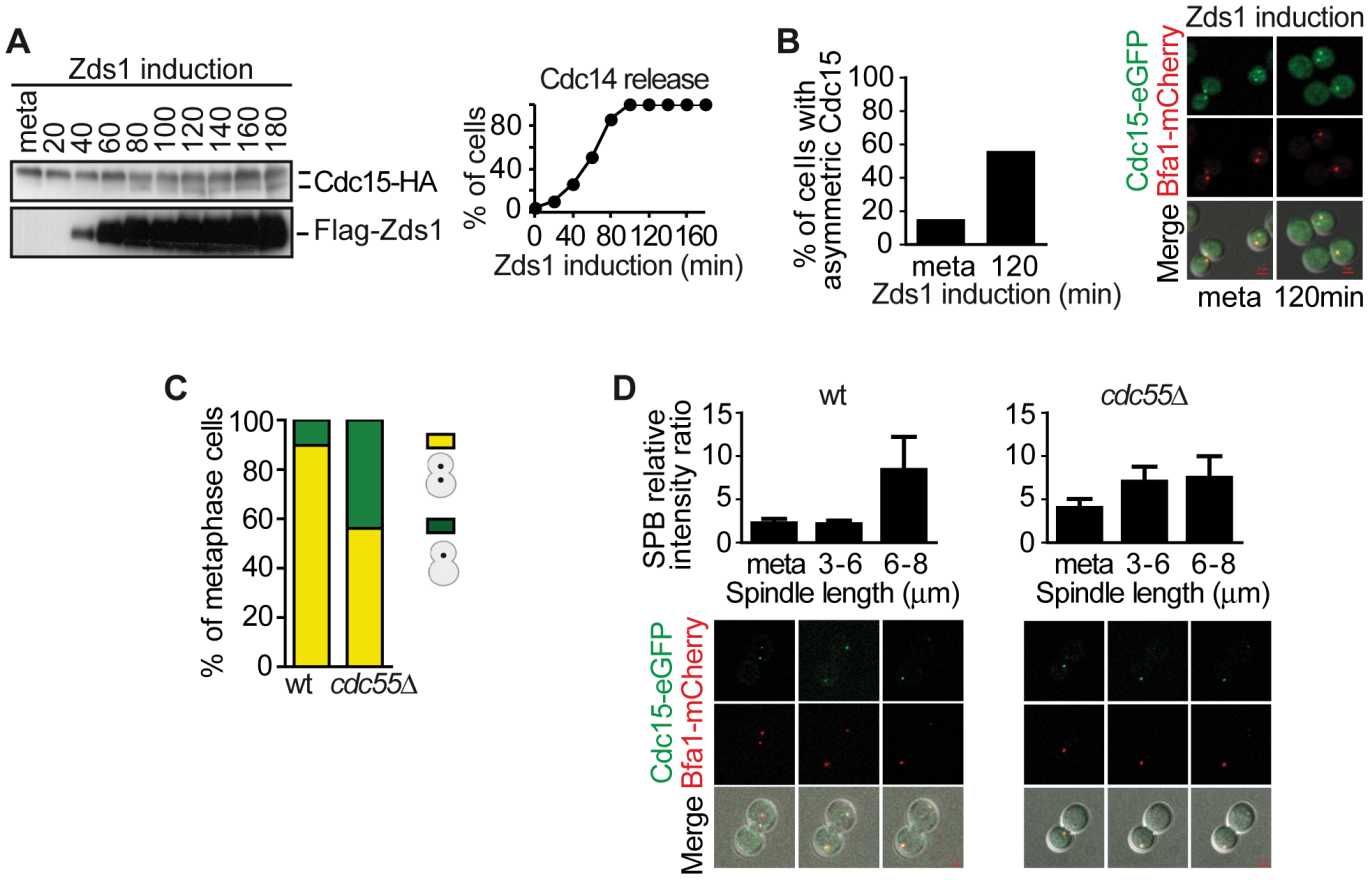
Downregulation of PP2A^Cdc55^ promotes Cdc15 activation. (A) Cdc15 is dephosphorylated upon Zds1-dependent inactivation of PP2A^Cdc55^. Strain Y603 (*MATa MET-CDC20 GAL1-Flag_3_-ZDS1 CDC14-Pk_9_ CDC15-HA_6_*) was arrested in metaphase by Cdc20 depletion and galactose was added to induce Zds1 expression. Cdc15 phosphorylation and Zds1 expression levels were analyzed by western blot. (B) Zds1-dependent inactivation of PP2A^Cdc55^ induces Cdc15 asymmetric localization. Strain Y1014 (*MATα MET-CDC20 GAL1-Flag_3_-ZDS1 CDC14-Pk_9_ CDC15-eGFP BFA1-mCherry*) was arrested in metaphase by Cdc20 depletion and Zds1 expression was induced. At least 50 cells were scored for each strain. (C) Cdc55 deletion causes premature Cdc15 asymmetric localization in metaphase. Strains Y984 (*MATa MET-CDC20 CDC14-myc_9_ CDC15-eGFP BFA1-mCherry*) and Y966 (as Y984, but *cdc55*Δ) were arrested in metaphase by Cdc20 depletion and the percentage of cells with asymmetric Cdc15-eGFP was quantified. At least 50 cells were scored for each strain. (D) Cdc15 premature asymmetric localization in the absence of Cdc55 in a metaphase-to-anaphase transition. The same strains as in (C) were released into a synchronous anaphase by Cdc20 depletion and reintroduction and followed by time-lapse microscopy (n = 14 for WT; n = 10 for *cdc55*Δ). Scale bar, 2 µm.

Given that PP2A^Cdc55^ counteracts Bfa1 phosphorylation in metaphase, and upon downregulation of PP2A^Cdc55^ in anaphase Bfa1 becomes hyperphosphorylated and located asymmetrically to the dSPB, the question arises as to why the exit from mitosis and MEN activation are not accelerated in *cdc55*Δ cells. It has recently been proposed that increased residence time of Tem1 on SPBs leads to premature Cdc15 loading but not to premature entry into anaphase [39]. To investigate whether something similar occurs in *cdc55*Δ cells, we examined Cdc15 and Dbf2-Mob1 activation. Cdc15 kinase is dephosphorylated and activated by early released Cdc14 from the nucleolus. Cells were arrested in metaphase by Cdc20 depletion and galactose was added to induce Zds1 ectopic expression (PP2A^Cdc55^ inactivation). Cdc15 phosphorylation status was analyzed by western blot (Figure 5A). Upon Zds1 induction, Cdc15 was dephosphorylated consistently with the result showed shown in Figure 3A, which implies that Cdc15 is dephosphorylated in the absence of PP2A^Cdc55^ activity. To characterize these cells further we checked the localization of Cdc15 on the SPBs upon Zds1-dependent PP2A^Cdc55^ inactivation. After Zds1 induction in metaphase-arrested cells, Cdc15-eGFP asymmetrically located onto the dSPB in 53% of the cells compared with 12% of non-induced cells (Figure 5B). We also studied Cdc15 localization in *cdc55*Δ cells in a metaphase-to-anaphase transition. Interestingly, in *cdc55*Δ cells Cdc15-eGFP was prematurely asymmetrically located onto the dSPB at metaphase in 44% of the cells (Figure 5C). In wild-type cells, Cdc15-eGFP was detected on the dSPB when cells entered anaphase, as reported previously [14], [16], [17], [38], [52], and as confirmed by the SPBs ratios (Figure 5D). In wild-type cells, the SPB ratio increased in anaphase cells with spindle lengths >6 µm, while in *cdc55*Δ mutant cells the ratio was already high at metaphase, indicative of asymmetric Cdc15 localization. Similar results were obtained with cells synchronized in G1 by α-factor (Figure S5). Therefore, upon inactivation of the PP2A^Cdc55^, Cdc15 was prematurely dephosphorylated and loaded onto the dSPB. These results suggest that in the absence of PP2A^Cdc55^ activity Tem1 is active and the MEN activation signal is transduced up to Cdc15.

**Figure 6:**
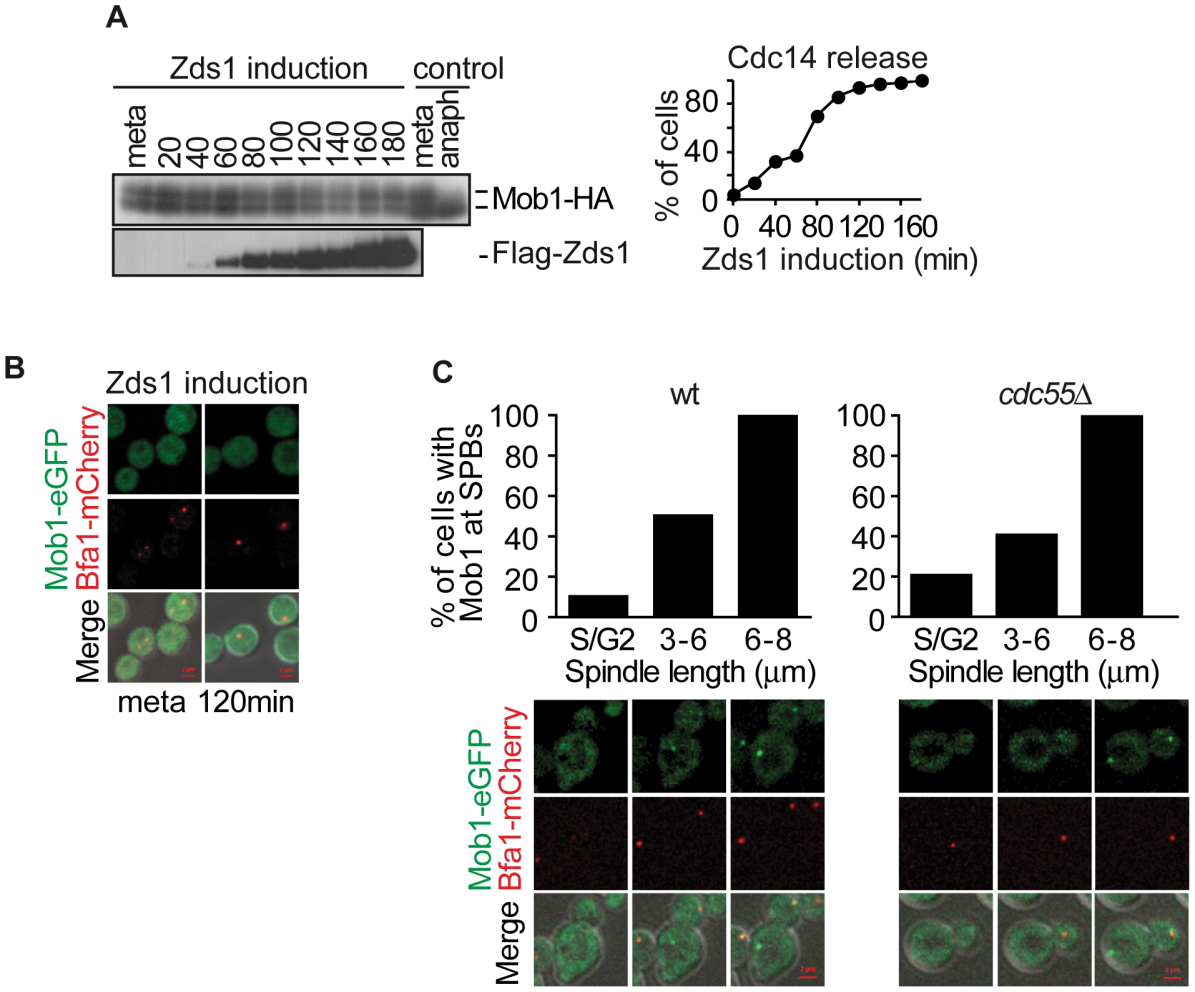
PP2A^Cdc55^ inactivation is insufficient to induce Dbf2–Mob1 activation. (A) Mob1 phosphorylation is not affected by PP2A^Cdc55^ downregulation after Zds1 induction. Strain Y807 (*MATα MET-CDC20 GAL1-Flag_3_-ZDS1 CDC14-Pk_9_ MOB1-HA_6_*) was arrested in metaphase by Cdc20 depletion and Zds1 expression was induced. Mob1 phosphorylation and Zds1 expression levels were analyzed by western blot. Anaphase (anaph) sample from a synchronous culture served as a Mob1 dephosphorylation control. (B) Mob1 is not loaded onto the SPB upon Zds1-dependent inactivation of PP2A^Cdc55^. Strain Y913 (*MATα MET-CDC20 GAL1-Flag_3_-ZDS1 CDC14-Pk_9_ BFA1-mCherry MOB1-eGFP*) was arrested in metaphase by Cdc20 depletion and galactose was added to induce Zds1 overexpression. At least 50 cells were scored for each strain. (C) Mob1 loading onto the SPB is restricted to anaphase in the absence of Cdc55. Strains Y1023 (*MATa MET-CDC20 CDC28F19 CDC14-myc_9_ MOB1-eGFP BFA1-mCherry*) and Y1076 (as Y1023, but *cdc55*Δ) were released into a synchronous cell cycle after α-factor arrest in G1 and followed by time-lapse microscopy (n = 10 for WT; n = 10 for *cdc55*Δ). Scale bar, 2 µm.

Dbf2–Mob1 localizes to both SPBs during anaphase [52], [53], which is important, though not sufficient, for Dbf2 kinase activity [52]. Dbf2 phosphorylation levels are inversely associated with its kinase activity [52]. Cdk1 is also known to phosphorylate and inhibit Mob1 [29]. Therefore, Dbf2–Mob1 phosphorylation levels are a good marker of Dbf2–Mob1 activity. For this reason, we next studied the Mob1 phosphorylation levels upon Zds1-dependent PP2A^Cdc55^ inactivation. Cells were arrested in metaphase by Cdc20 depletion and Zds1 ectopic expression was induced (Figure 6A). Strikingly, Mob1 was not dephosphorylated upon Zds1 induced-PP2A^Cdc55^ inactivation. Upon Zds1 overexpression, Cdk1–Clb2 kinase is still active and maintains Mob1 phosphorylated and inactive. Consistent with this experiment, we were not able to detect any Mob1-eGFP signal in the SPBs upon Zds1 induction in metaphase-arrested cells (Figure 6B). Moreover, the Mob1-eGFP signal in SPBs increased in anaphase in a similar way in wild-type and *cdc55*Δ mutant cells (Figure 6C). Therefore, we can conclude that in the absence of PP2A^Cdc55^ activity, Cdc15 is untimely dephosphorylated and loaded onto the dSPB, but there is no premature mitotic exit because Dbf2-Mob1 remains inactive.

### Concerted MEN regulation by Bub2–Bfa1 and Cdk1–Clb2

**Figure 7:**
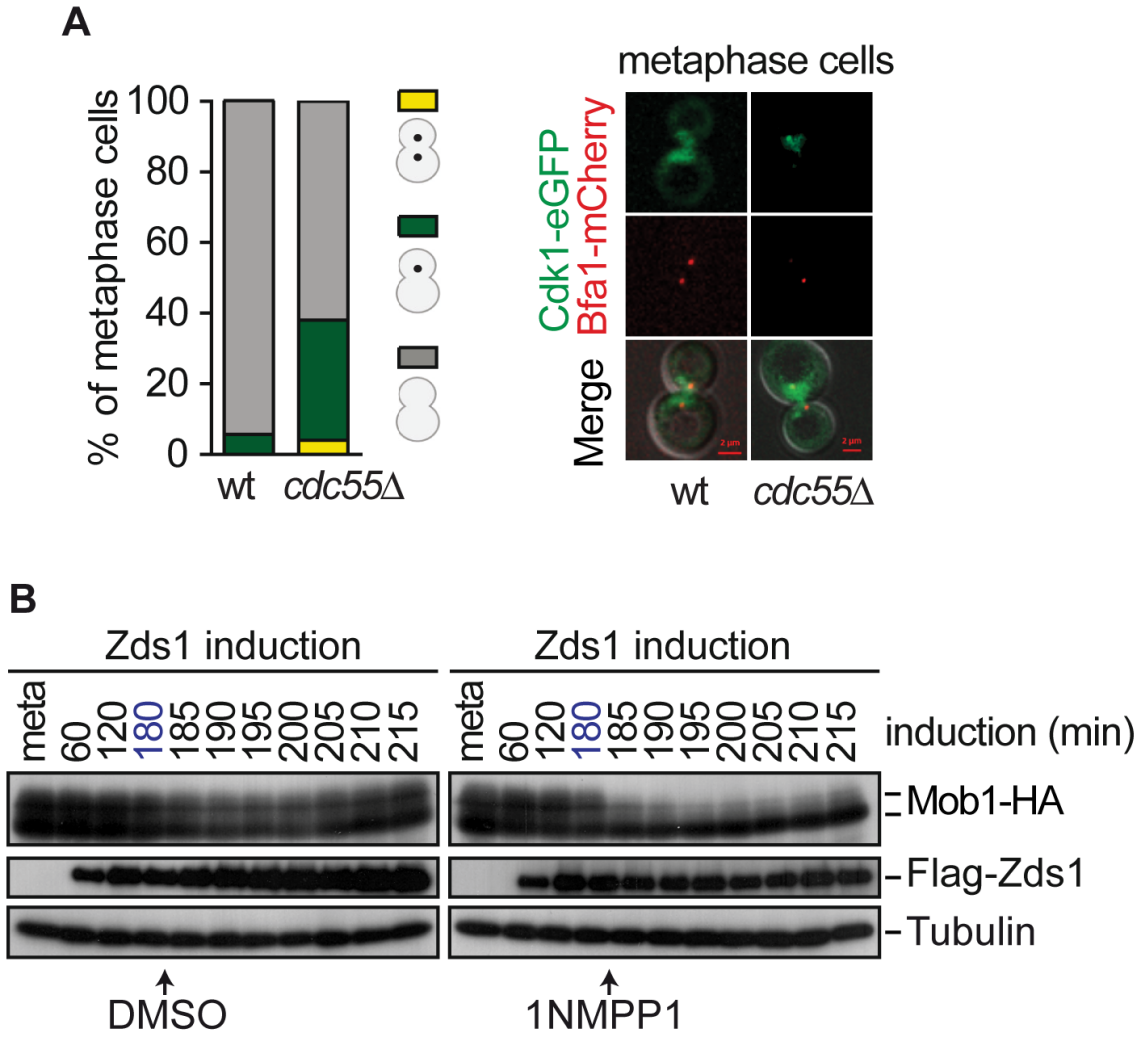
Cdk1– Clb2 inhibitory signal restrains MEN activation until anaphase. (A) Premature Cdk1-Clb2 localization at the SPB during metaphase in the absence of Cdc55. Strains Y985 (*MATa MET3-CDC20 CDC28F19 CDC14- myc_9_ Cdk1-eGFP BFA1-mCherry*) and Y967 (as Y985, but *cdc55*Δ) were arrested in metaphase by Cdc20 depletion. At least 50 cells were scored for each strain. (B) Mob1 dephosphorylation upon Cdk1 inhibition. Strain Y1032 (*MATa MET3-CDC20 CDC28-as1 GAL1-Flag_3_-ZDS1 MOB1-HA_6_*) were arrested in metaphase by Cdc20 depletion and Zds1 was induced. After 180 min of Zds1 induction, 1 µM of 1NM-PP1 drug was added to inhibit Cdk1 to half of the culture and DMSO was added to the other half as a control. Scale bar, 2 µm.

Non-phosphorylated Cdc15 is recruited to the mSPB in early anaphase and then helps load Cdk1 and Dbf2–Mob1 onto this SPB. It has been suggested that the close vicinity of the proteins at the mSPB leads to Cdc15 and Mob1 phosphorylation by Cdk1. Phosphorylated Cdc15 then dissociates from the mSPB and become asymmetrically localized to the dSPB [29]. Since Cdc15 shows premature asymmetric localization at the dSPB, we next investigated whether Cdk1 binds to the mSPB upon PP2A^Cdc55^ inactivation. If MEN activity were inhibited during metaphase by Cdk1–Clb2 in *cdc55*Δ cells, we would expect to detect Cdk1-eGFP on the mSPB. Indeed, in *cdc55*Δ cells metaphase-arrested by Cdc20 depletion Cdk1-eGFP was prematurely visualized on the mSPB in 38% of the cells (Figure 7A). However, Cdk1-eGFP could only be detected on the mSPB during anaphase in wild-type cells. It is noted that the Cdk1-eGFP signal always opposes the Bfa1-mCherry signal. This result is consistent with the premise that Dbf2–Mob1 inhibition by Cdk1–Clb2 is predominant in *cdc55*Δ mutant cells in metaphase and restrains these cells from a premature exit from mitosis.

The above results led us to hypothesize that alleviation of the Bfa1–Bub2 inhibitory MEN signal is insufficient to promote premature exit from mitosis in *cdc55*Δ cells, and that Cdk1–Clb2 inhibitory MEN signal is the additional mechanism that restrains MEN activity until anaphase. We have observed that Mob1 was not dephosphorylated upon Zds1-induced-PP2A^Cdc55^ inactivation despite Cdc14 being released from the nucleolus. Therefore, the Cdk1–Clb2 phosphorylation event towards Mob1 predominates over the action of the phosphatases Cdc14 and PP2A^Cdc55^. To test this hypothesis, we inactivated PP2A^Cdc55^ by Zds1 induction to promote Cdc14 release, and inhibited Cdk1 by adding 1NM-PP1 in cells bearing the ATP analog-sensitive Cdk allele, *cdc28-as1*. We arrested cells in metaphase by Cdc20 depletion and induced Zds1 by galactose addition. After 180 min of Zds1 induction, when 80% of cells had released Cdc14 from the nucleolus (indicative of PP2A^Cdc55^ inactivation), the drug 1NM-PP1 was added to inhibit Cdk1. Remarkably, upon Cdk1 inactivation Mob1 was rapidly dephosphorylated (Figure 7B). Mob1 phosphorylation status was unchanged in the control cells without the drug. We can conclude that cells can prematurely activate Dbf2–Mob1 after removal of the Cdk1–Clb2 inhibitory signal. This result demonstrates that Cdc14 release from the nucleolus is not enough to promote MEN activation, and additional mechanisms like Cdk1–Clb2 and Bub2–Bfa1 inhibitory signals ensure proper progression through mitosis.

## Discussion

The means by which chromosome segregation is coordinated with sequential Cdk1 inactivation steps during mitosis is a subject of great interest. It is known that separase activation triggers both chromosome segregation and FEAR-Cdc14 release. However, we do not fully understand the separate and specific regulation of the FEAR and MEN components or how these pathways are coordinated during anaphase. Downregulation of PP2A^Cdc55^ phosphatase at anaphase onset facilitates Cdk1-dependent Net1 phosphorylation, which provides the first wave of Cdc14 release [9]. PP2A^Cdc55^ phosphatase downregulation could be involved in other processes, such as facilitating MEN activation in anaphase. In the present study, we determined that PP2A^Cdc55^ regulates Bfa1 phosphorylation: Bfa1 is hyperphosphorylated in *cdc55*Δ cells, upon inhibition of PP2A^Cdc55^ by Zds1 overexpression or by inactivation of the catalytic PP2A subunits; and overexpression of Cdc55 avoids Bfa1 phosphorylation in G2/M. Moreover, Bfa1 and Cdc55 physically interact, suggesting that Bfa1 is likely to be an *in vivo* substrate of PP2A^Cdc55^. In this way, PP2A^Cdc55^ downregulation by separase would unlock mitotic exit, initiating FEAR-Cdc14 release and the MEN pathway.

PP2A^Cdc55^ seems to be specific to Bfa1, since Bfa1 phosphorylation is not affected in *rts1*Δ deletion mutants [41]. Moreover, upon Zds1 pull-down and mass-spectrometry analysis Cdc55 was identified but Rts1 was not detected [54], and Bfa1 is properly phosphorylated upon Zds1 overexpression in an *rts1*Δ mutant (data not shown). Therefore, a contribution of Zds1 to the PP2A^Rts1^ complex is not expected.

In the normal cell cycle, Bfa1 inactivation depends on Cdc5 phosphorylation. Although Bfa1 may be a substrate for other kinases that have not yet been described, our results indicate that PP2A^Cdc55^ counteracts Bfa1 Cdc5-dependent phosphorylation. Previous results have already shown that PP2A^Cdc55^ can counteract Cdc5-dependent phosphorylation [55]. Therefore, PP2A^Cdc55^ is not only a Cdk1–Clb2-counteracting phosphatase, but is also able to dephosphorylate Cdc5 targets.

Our results indicate that Mob1 phosphorylation is also regulated by the phosphatase PP2A^Cdc55^. Mob1 is phosphorylated [29], [56] and inhibited by Cdk1 [29], and is dephosphorylated by Cdc14 [29] and PP2A^Cdc55^ (our results). Progressive quantitative changes of the Cdc14/Cdk activity ratio during the course of mitotic exit cause dephosphorylation of individual substrates at distinct thresholds [42]. Several observations suggest that Mob1 might be a late Cdc14 substrate that maintains its Cdk1-phosphorylation until late anaphase: (a) Dbf2–Mob1 dephosphorylation occurs in mid/late anaphase ([52] and Figure 6B); (b) Mob1 is phosphorylated even upon PP2A^Cdc55^ inactivation in which Cdc14 has already been released from the nucleolus (Figure 5A); and (c) during anaphase, PP2A^Cdc55^ phosphatase activity is reduced [9] helping to keep Mob1 phosphorylated. Later, upon Cdk1 inhibition Cdc14, and probably also PP2A^Cdc55^, will dephosphorylate Mob1, thereby allowing full MEN activation.

Premature Bfa1 phosphorylation observed after PP2A^Cdc55^ inactivation does not entail premature MEN activation and mitotic exit. Cdc15 dephosphorylation patterns have often been used as a marker of MEN activity. However, we observed that Cdc15 is prematurely dephosphorylated and loaded onto the dSPB in *cdc55*Δ mutant cells and after Zds1-induced PP2A^Cdc55^ inactivation. This is consistent with Cdc14 being released from the nucleolus prematurely during metaphase in *cdc55*Δ mutant cells. Despite Cdc15 being properly recruited to the dSPB, our results suggest that Cdc15 is not totally active as a kinase, since Dbf2-Mob1 is not recruited to the SPBs after PP2A^Cdc55^ inactivation. In accordance with this, Cdc15 dephosphorylation never reaches the levels of wild-type cells in anaphase indicating that it is not fully active. In addition, Mob1 is not dephosphorylated upon PP2A^Cdc55^ inactivation by Zds1 induction (despite Cdc14 being released), further confirming that Dbf2-Mob1 is not active in this condition. The study of late anaphase events confirmed the previous results. Cdh1, which is a late Cdc14 substrate, is not properly dephosphorylated, resulting in a defective APC^Cdh1^ complex. Thus, APC^Cdh1^ substrates such as Cdc5 Polo kinase and Clb2 are not efficiently degraded. These results led us to propose that there is an inhibitory input into the MEN cascade downstream of Bfa1 that acts as a break when Bfa1 is prematurely phosphorylated, thereby avoiding the untimely full activation of MEN. In fact, Cdk1-dependent Cdc15 inhibition has been postulated before [28], and previous publications by our group have presented a mathematical model to describe the negative regulation of Cdc15 by Cdk–Clb2 [9], [57], [58]. The mutual regulation of Cdk–Clb2 and Cdc15 has been described more recently [29]. These authors also demonstrated that Cdk1 phosphorylates the Mob1 protein to inhibit the activity of Dbf2–Mob1 kinase. Finally, there is genetic evidence of a concerted action by Bfa1 and Clb2 when Tem1 is accumulated in the SPBs, [39]. We found that Cdk1-eGFP was prematurely located on the mSPB in metaphase *cdc55Δ* cells, as would be expected if MEN activity were inhibited by Cdk1–Clb2. Moreover, Mob1 was rapidly dephosphorylated upon Cdk1 inactivation after Zds1-dependent Cdc14 release. The above results led us to postulate that alleviation of the Bub2–Bfa1 inhibitory MEN signal is not enough to produce the premature exit from mitosis observed in *cdc55*Δ cells, and that the Cdk1–Clb2 inhibitory MEN signal is the additional mechanism that restrains MEN activity until anaphase. In this way, cells would initiate FEAR and MEN at anaphase onset upon PP2A^Cdc55^ downregulation, but Cdk1–Clb2 would impose a break for MEN activation, and decreasing Cdk1 activity would be the mechanism that sequentially activates both pathways.

APC^Cdh1^ and Bub2–Bfa1 are both required to resequester Cdc14 into the nucleolus after mitotic exit [36], [52], [59]. APC^Cdh1^-dependent Cdc5 degradation is important to return Cdc14 to the nucleolus at the correct time, but it is not essential, since Cdc14 is resequestered into the nucleolus, albeit with a delay in cells lacking *CDH1* [59]. Moreover, cells lacking both *BUB2* and *CDH1* eventually resequester Cdc14 into the nucleolus, suggesting that additional mechanisms regulate Cdc14 resequestration. PP2A^Cdc55^ reactivation during late anaphase could be one of these additional mechanisms involved in the resequestration of Cdc14 into the nucleolus. We have demonstrated here that Cdh1 is not fully active since Cdc5 and Clb2 degradation are impaired in *cdc55Δ* mutant cells. Net1 dephosphorylation is required to reassociate to Cdc14 and resequester it into the nucleolus; Bfa1 dephosphorylation is needed to inactivate MEN and helps to return Cdc14 to the nucleolus. In cells lacking *CDC55*, Net1 is hyperphosphorylated for longer [9], suggesting that PP2A^Cdc55^-dependent dephosphorylation in late anaphase is important for properly resequestering Cdc14 into the nucleolus. Nevertheless, the *cdc55Δ* mutant cells exit from mitosis, despite their slower kinetics in resequestering Cdc14 to the nucleolus. Sic1 is accumulated at the correct time in the cells lacking *CDC55* and its level remains constant for longer periods. Sic1 accumulation is probably sufficient to inhibit Cdk1 and to exit from mitosis in the absence of Cdc55. This is consistent with *GAL-SIC1*-db being sufficient to drive exit from mitosis in MEN mutants [53], [60].

**Figure 8:**
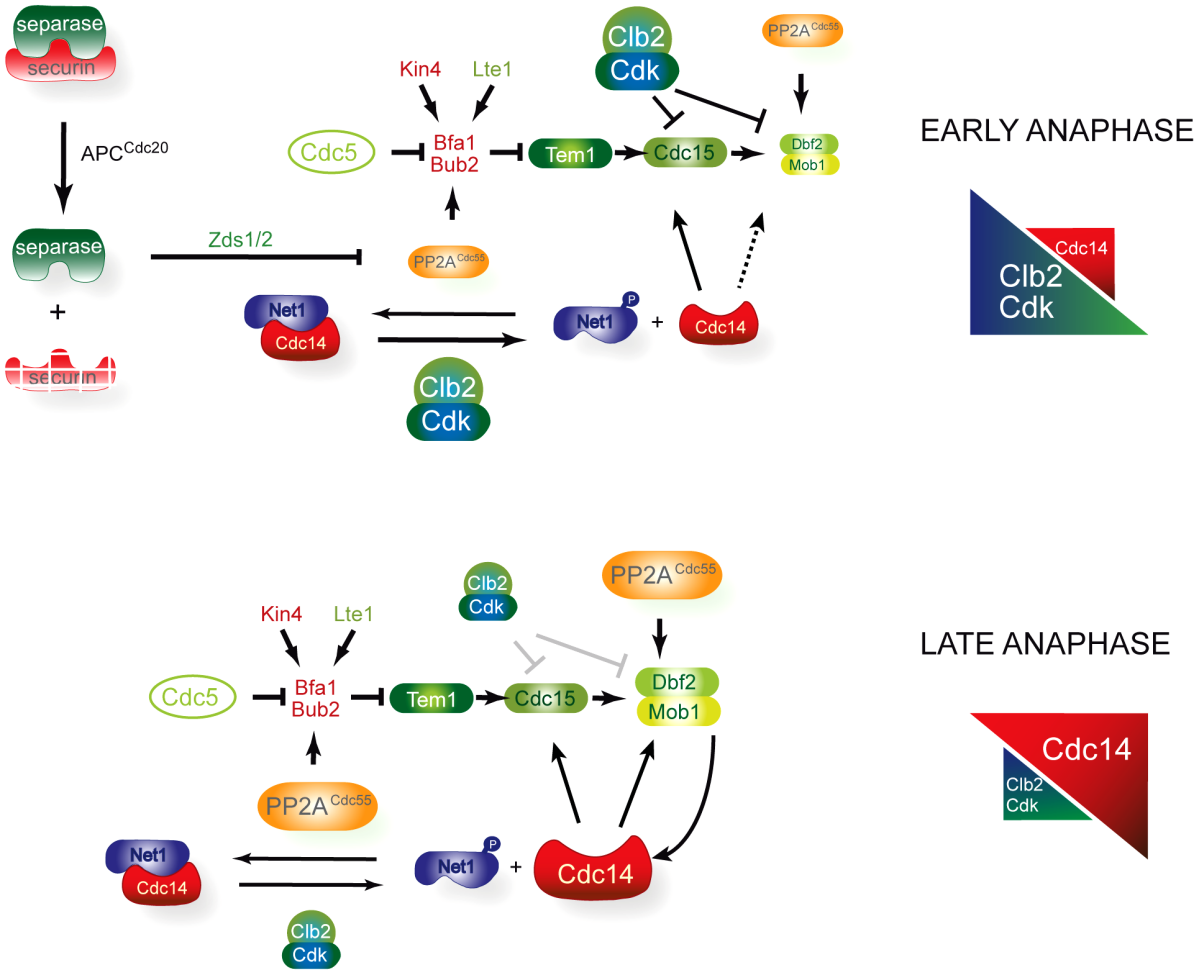
A model for MEN regulation by PP2A^Cdc55^. In early anaphase, FEAR-induced inactivation of the PP2A^Cdc55^ promotes the first wave of Cdc14 release and contributes to the accumulation of the Cdc5-phosphorylated form of Bfa1. In this way, FEAR alleviates the inhibitory signal on Tem1 by promoting the Bfa1 phosphorylation and stimulates Cdc15 activity through the FEAR-induced Cdc14 released. However, the downstream MEN kinase Dbf2–Mob1 is kept inactive by Cdk1–Clb2 phosphorylation, restraining MEN activity. Mob1 phosphorylation is invariable despite the progressive decrease in Cdk1 activity because the counteracting phosphatase PP2A^Cdc55^ is also downregulated in early anaphase. In late anaphase, the increase in Cdc14 activity and the decrease in Cdk1–Clb2 activity alleviate the Cdk1 inhibitory signal towards Dbf2–Mob1 and the MEN is fully active.

Our model, based on the current findings and those of previous published work, is summarized in Figure 8. In early anaphase, FEAR-induced inactivation of the PP2A^Cdc55^ promotes the first wave of Cdc14 release from the nucleolus and contributes to the accumulation of the Cdc5-phosphorylated form of Bfa1. In this way, FEAR promotes the activation of two important MEN components: (1) PP2A^Cdc55^ downregulation facilitates Bfa1 inactivation, allowing Tem1 activation; and (2) the FEAR-induced Cdc14 released dephosphorylates Cdc15. Bfa1 asymmetric localization on the dSPB is induced upon Bfa1 phosphorylation and is important for the proper recruitment of MEN components to the dSPB. In addition, Tem1 binds to the mSPB and recruits Cdc15. Cdc15 itself recruits Cdk1 and Dbf2-Mob1 to the mSPB. In turn, Cdk1 phosphorylates Cdc15, which is dissociated from the mSPB and becomes asymmetrically located on the dSPB. At the same time, Cdk1 phosphorylates and inhibits Dbf2–Mob1. Our results indicate that Mob1 phosphorylation is also regulated by the phosphatase PP2A^Cdc55^, helping to keep Dbf2–Mob1 inactive in early anaphase. In this way, Mob1 phosphorylation is constant despite the progressive decrease in Cdk1 activity because the counteracting phosphatase PP2A^Cdc55^ is also downregulated. In late anaphase, the increase in Cdc14 activity and the decrease in Cdk1 activity alleviate the Cdk1 inhibitory signal towards Dbf2–Mob1, and the MEN is fully active. Upon its reactivation, PP2A^Cdc55^ will also help keep Mob1 dephosphorylated.

Exit from mitosis is regulated by a conserved signalling pathway in *Schizosaccharomyces pombe* called the septation initiation network (SIN) (reviewed in [61], [62]). Physical interaction between fission yeast Pab1 (B-regulatory subunit of PP2A in *S. pombe*) and Sid2 (Mob1 orthologue) has recently been described [63]. PP2A-Pab1 regulates SIN activity at different levels, suggesting a conserved function for PP2A^Cdc55^ in regulating the MEN and SIN pathways. Although the SIN is analogous to the MEN in budding yeast, its main role is to coordinate cytokinesis. However, in *S. cerevisiae*, the MEN also goes beyond mitotic exit and regulates cytokinesis. Several MEN components localized to the bud neck and were found to be required for the contraction of the actin-myosin ring [17], [52], [53], [64], [65]. More recent evidence suggests that Dbf2–Mob1 regulates the cytokinetic components Chs2, Hof1 and Inn [66]–[68]. The core signalling elements of the MEN/SIN pathway are composed of members that are highly conserved in a range of species from yeast to humans (reviewed in [69]). The MEN, SIN and Hippo pathways in *Drosophila* and vertebrates share elements of the Cdc15-like kinases (Sid1 in *S. pombe* and MTS1/2 in mammals) and Dbf2-Mob1-like kinases (Sid2–Mob1 in *S. pombe* and LATS1/2-MOB1 in mammals) and the Cdc14 protein phosphatase family (Clip in *S. pombe* and Cdc14 in mammals). Some studies suggest that the Hippo pathway plays a role in regulating mitotic exit and cytokinesis, although the mechanism is not yet fully understood. Our studies of MEN regulation will contribute to our understanding of MEN-related pathways in other organisms. It will be interesting to discover whether MEN regulation by PP2A^Cdc55^ is conserved among higher eukaryotes.

## Materials and Methods

### Yeast strains, plasmids and cell cycle synchronization procedures

All yeast strains used in this study were derivatives of W303. Epitope tagging of endogenous genes and gene deletions were performed by gene targeting using polymerase chain reaction (PCR) products. Endogenous *CDC55* was N-terminal-tagged as previously described [9]. *CDC28^Y19F^* mutant strains were created by integration and loop-out, via 5-FOA (5-fluoroorotic acid) selections. Zds1 in cells that had been arrested in metaphase by Cdc20 depletion was ectopically expressed as previously described [10]. Cell synchronization using α-factor and metaphase arrest by Cdc20 depletion and entry into synchronous anaphase by Cdc20 reinduction were also performed as previously described [70].

### Image analysis and time-lapse experiments

For the Zds1-induction experiments cells were fixed in absolute ethanol throughout the experiment and rehydrated in minimum media before visualization. At least 50 cells were used to quantify the SPB ratios. For time-lapse microscopy, cells were incubated in minimum media throughout the experiment in an environmental chamber, and images were acquired every 5 minutes. A Zeiss Axio Observer Z1 inverted epifluorescence microscope with Apotome system equipped with an HXP 120C fluorescent lamp and a Carl Zeiss Plan-Apochromat 63× N.A 1.40 oil objective. Filters used were EGFP (EX BP 470/40, BS FT 495, EM BP 525/50) and Cy3 (EX BP 550/25, BS FT 570, EM BP 605/70). Z-stacks at 1-µm intervals were taken for each fluorescent channel and projected onto a single image per channel. SPB ratios were quantified as the signal intensity ratio between the two SPBs (dSPB relative intensity/mSPB relative intensity). SPB ratios less than or equal to 2 or greater than 2 were considered to represent symmetric or asymmetric localization, respectively. We used ZEN software for image acquisition and quantitative analysis of fluorescent microscopy (signal intensity and spindle length).

### SILAC labeling and phosphoproteomic analysis

Stable isotopes of yeast cells were labeled and protein extracts prepared as previously described [71]. Cells were grown in minimum media containing either 100 mg/L arginine and 100 mg/L lysine or 100 mg/L ^13^C_6_-arginine and 100 mg/L ^13^C_6_-lysine (Cambridge Isotope Laboratories Inc.). Protein extracts were prepared by mechanical lysis using glass beads. Approximately 250 µg of the mixed heavy/light protein sample were processed for in-solution digestion as previously described [72]. Phosphopeptides were enriched by sequential elution from IMAC (SIMAC) as previously described [71]. Peptides were analyzed by LC-MS/MS using an LTQ-Orbitrap Velos mass spectrometer (Thermo Scientific). Peptides were identified using MASCOT software and SILAC quantification was done with ProteomeDiscoverer 1.3 Software (Thermo Scientific).

### Immunoprecipitation and Cdc14 phosphatase assay

The immunoprecipitation assay was performed as described [10], but using 2×10^9^ cells for Mob1 and 3.2×10^10^ cells for Bfa1. Protein extracts were prepared by mechanical lysis using glass beads. The clarified extracts were incubated with antibody, and the immunocomplexes were adsorbed onto magnetic protein-A Dynabeads® (Life Technologies). The beads were washed in extraction buffer and the protein-bound fraction eluted with SDS-PAGE loading buffer. The antibody used for immunoprecipitation was the α-Pk clone SV5-Pk1 (Serotec). For the phosphatase assays, Pk epitope-tagged Cdc14 was immunopurified on protein-A Dynabeads as above, and the beads were washed with phosphatase buffer (50 mM Tris-HCl pH 7.0, 1% BSA, 150 mM NaCl, 2 mM MnCl_2_). Reaction mix (phosphatase buffer containing 34 µM of DiFMUP) was added to the beads and incubated at 30°C for 2 min. Reactions were terminated by adding 200 mM sodium carbonate and the fluorescence of the samples was determined at 365 nm (absorption wavelength) and 455 nm (emission wavelength). Cdc14 recovered on the beads was quantified by western blot using an IRdye 800 coupled secondary antibody and the Odyssey Infrared Imaging System (Li-COR Biosciences). Phosphatase-specific activity was calculated as fluorescence units/protein levels and represented as arbitrary units.

### Other techniques

Protein extracts for western blots were obtained by NaOH or TCA protein extraction. Bfa1 and Cdc15 western blots where done using 10% protein gel at a 33.5∶0.3 acrylamide/bisacrylamide ratio. Antibodies used for western blots and immunofluorescence were the α-HA clone 12CA5 (Roche), α-HA clone 16B12 (Babco), Cdc14 (yE-17) sc-12045 (Santa Cruz Biotechnology), α-FLAG clone M2 (Sigma), α-Pk clone SV5-Pk1 (Serotec), Cdc5 (yC-19) sc-6733 (Santa Cruz Biotechnology), Sic1 (FL-284) sc-50441 (Santa Cruz Biotechnology), Clb2 (y-180) sc-907 (Santa Cruz Biotechnology), anti-tubulin clone YOL1/34 (Serotec) and anti-phosphoglycerate kinase (Life Technologies). The secondary antibodies were Cy3-labeled α-mouse (GE Healthcare), fluorescein-conjugated α-rat (Millipore) and Cy3-labeled α-goat (Jackson ImmunoResearch).

## Supporting Information

Figure S1 Separase-dependent inactivation of PP2A^Cdc55^ promotes Bfa1 phosphorylation. Strain Y532 (*MATa MET-CDC20 GAL1-Flag_3_-ESP1 CDC14-Pk_9_ BFA1-HA_6_*) was arrested in metaphase by Cdc20 depletion, and separase ectopic expression was induced by galactose addition. Bfa1 phosphorylation and Esp1 expression levels were analyzed by western blot. Cdc14 release was monitored by immunofluorescence as control of PP2A^Cdc55^ inactivation.
(TIF)

Figure S2 Cdc5 Polo-like kinase-dependent Bfa1 phosphorylation is required for Zds1-induced PP2A^Cdc55^ inactivation. Strains Y1034 (*MATa MET-CDC20 GAL1-Flag_3_-ZDS1 CDC14-Pk_9_ BFA1-HA_6_*) and Y1033 (as Y1034, but *cdc5Δ CDC5-14-HA_3_*) were arrested in metaphase by Cdc20 depletion and shifted to 37°C for 180 min before Zds1 induction. Bfa1 phosphorylation and Zds1 expression levels were analyzed by western blot. Cdc14 release was monitored by immunofluorescence.
(TIF)

Figure S3 Bfa1 phosphorylation observed upon PP2A^Cdc55^ inactivation does not depend on the kinase Kin4. Strains Y597 (*MATa MET-CDC20 GAL1-Flag_3_-ZDS1 CDC14-Pk_9_ BFA1-HA_6_*) and Y563 (as Y597 but *kin4*Δ) were arrested in metaphase by Cdc20 depletion and Zds1 ectopic expression was induced by galactose addition. Bfa1 phosphorylation and Zds1 expression levels were analyzed by western blot. Cdc14 release was monitored by immunofluorescence as control of PP2A^Cdc55^ inactivation.
(TIF)

Figure S4 Zds1 protein levels. Zds1-induction controls of experiments in Figs. 4B, 5B and 6B. Zds1 protein levels were analyzed by western blot.
(TIF)

Figure S5 Increased Cdc15 asymmetric localization in the absence of Cdc55 in a synchronous cell cycle after G1 release. Strains Y911 (*MATa CDC28F19 CDC14-myc_9_ CDC15-eGFP BFA1-mCherry*) and Y957 (as Y911, but *cdc55*Δ) were arrested at G1 with α-factor and released into a synchronous cell cycle. Time-lapse microscopy was performed as described in the Material and Methods.
(TIF)
