# Supplementary figures and images for: Dual Regulation of the Mitotic Exit Network (MEN) by PP2A-Cdc55 Phosphatase
Source: PLoS Genet. 2013 Dec 5;9(12):e1003966. doi: 10.1371/journal.pgen.1003966 (PMC3854864; doi:10.1371/journal.pgen.1003966)

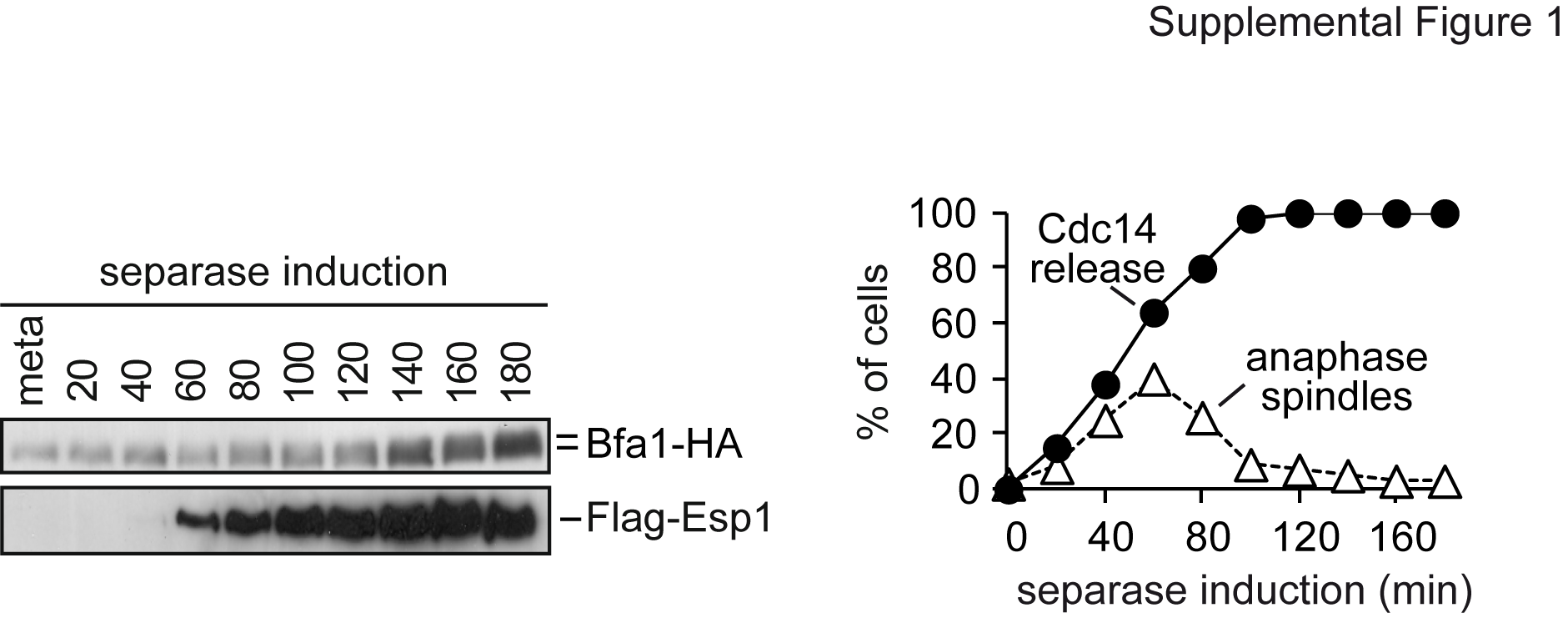

Supplement: Figure S1 — Separase-dependent inactivation of PP2ACdc55 promotes Bfa1 phosphorylation. Strain Y532 (MATa MET-CDC20 GAL1-Flag3-ESP1 CDC14-Pk9 BFA1-HA6) was arrested in metaphase by Cdc20 depletion, and separase ectopic expression was induced by galactose addition. Bfa1 phosphorylation and Esp1 expression levels were analyzed by western blot. Cdc14 release was monitored by immunofluorescence as control of PP2ACdc55 inactivation. (TIF) [file pgen.1003966.s001.tif]

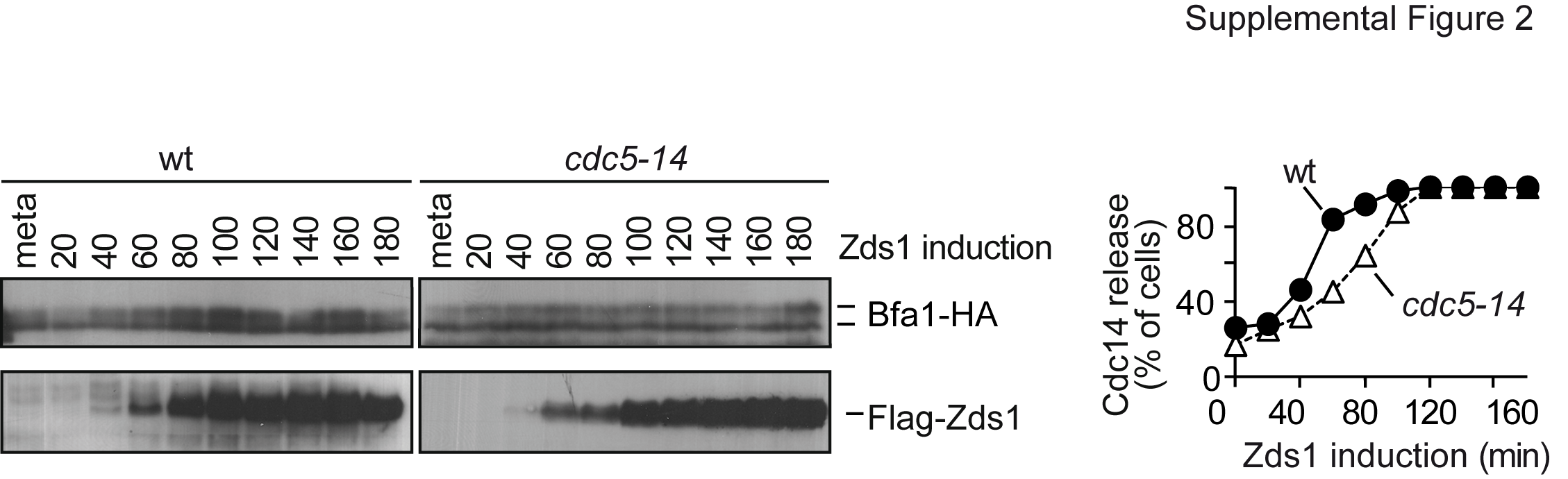

Supplement: Figure S2 — Cdc5 Polo-like kinase-dependent Bfa1 phosphorylation is required for Zds1-induced PP2ACdc55 inactivation. Strains Y1034 (MATa MET-CDC20 GAL1-Flag3-ZDS1 CDC14-Pk9 BFA1-HA6) and Y1033 (as Y1034, but cdc5Δ CDC5-14-HA3) were arrested in metaphase by Cdc20 depletion and shifted to 37°C for 180 min before Zds1 induction. Bfa1 phosphorylation and Zds1 expression levels were analyzed by western blot. Cdc14 release was monitored by immunofluorescence. (TIF) [file pgen.1003966.s002.tif]

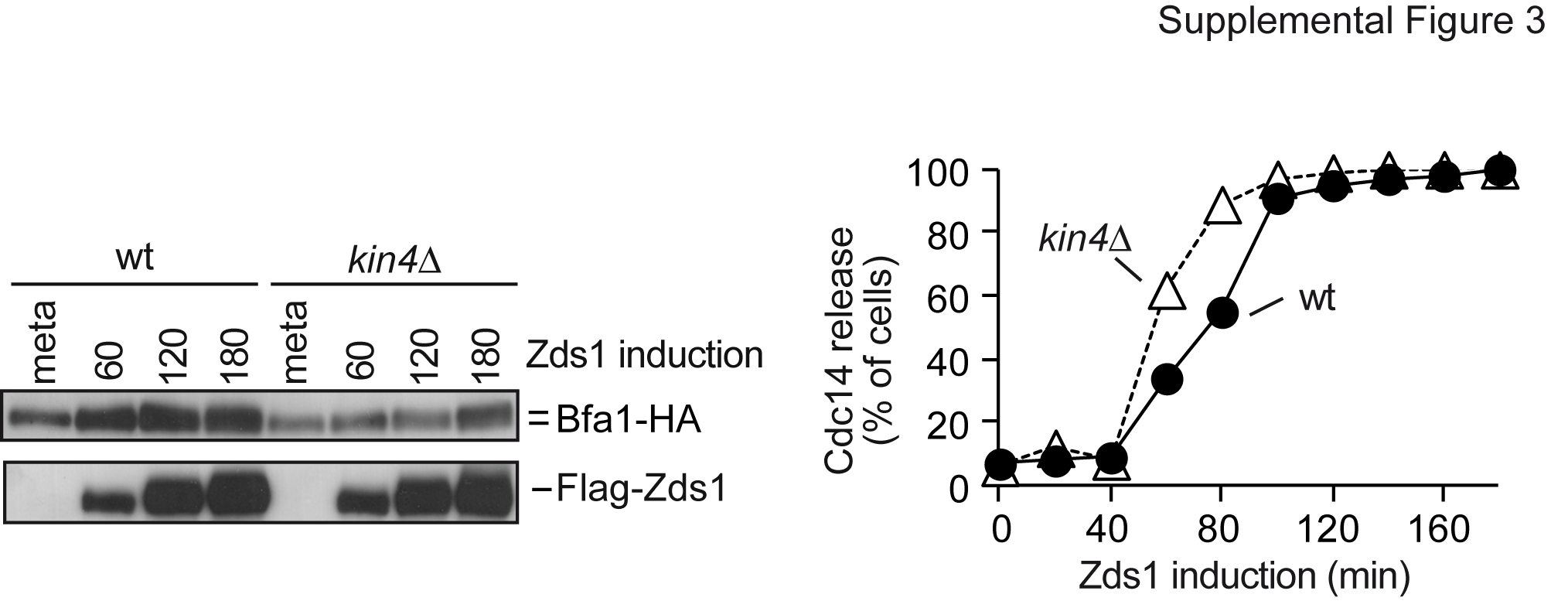

Supplement: Figure S3 — Bfa1 phosphorylation observed upon PP2ACdc55 inactivation does not depend on the kinase Kin4. Strains Y597 (MATa MET-CDC20 GAL1-Flag3-ZDS1 CDC14-Pk9 BFA1-HA6) and Y563 (as Y597 but kin4Δ) were arrested in metaphase by Cdc20 depletion and Zds1 ectopic expression was induced by galactose addition. Bfa1 phosphorylation and Zds1 expression levels were analyzed by western blot. Cdc14 release was monitored by immunofluorescence as control of PP2ACdc55 inactivation. (TIF) [file pgen.1003966.s003.tif]

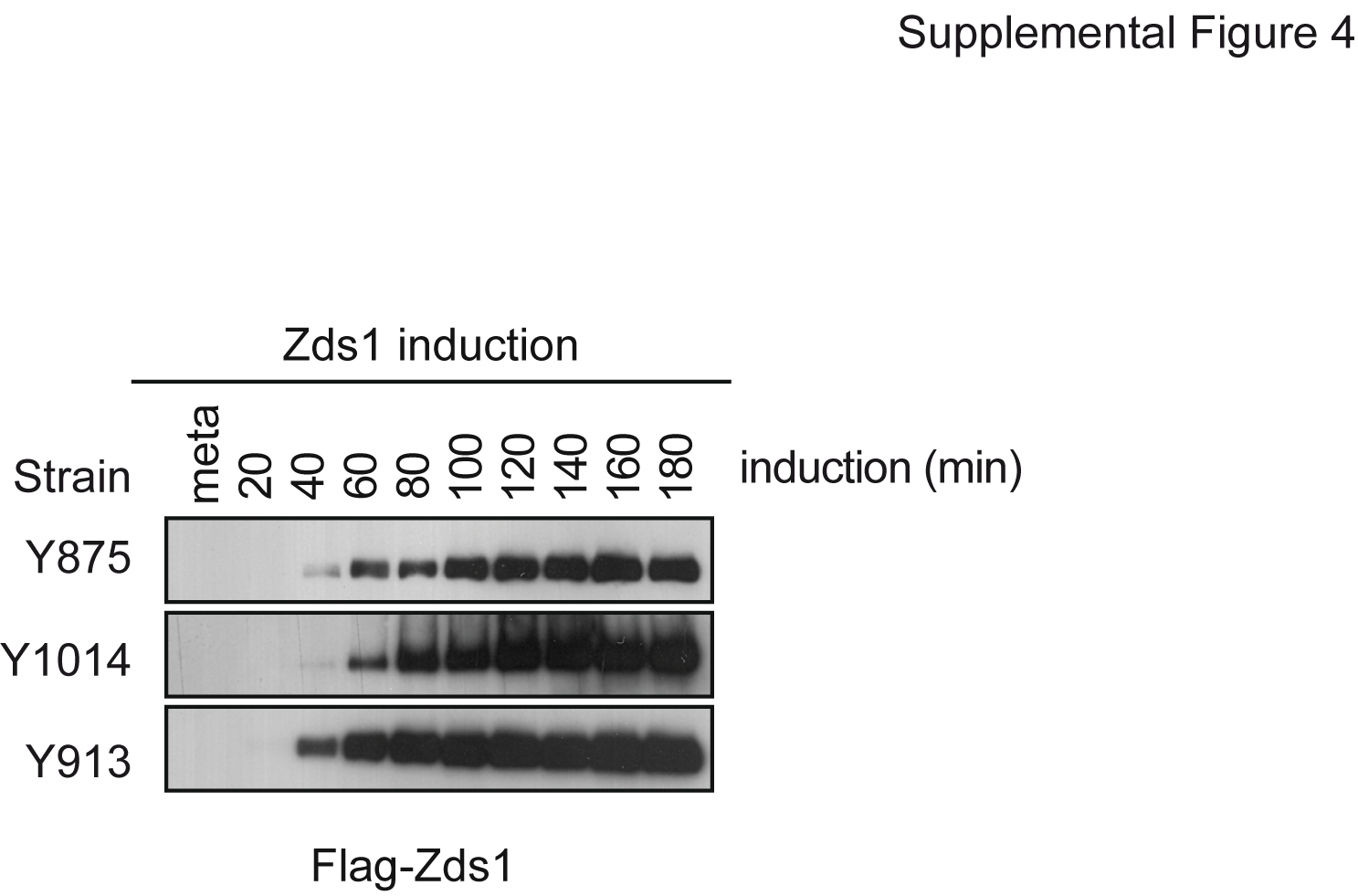

Supplement: Figure S4 — Zds1 protein levels. Zds1-induction controls of experiments in Figs. 4B, 5B and 6B. Zds1 protein levels were analyzed by western blot. (TIF) [file pgen.1003966.s004.tif]

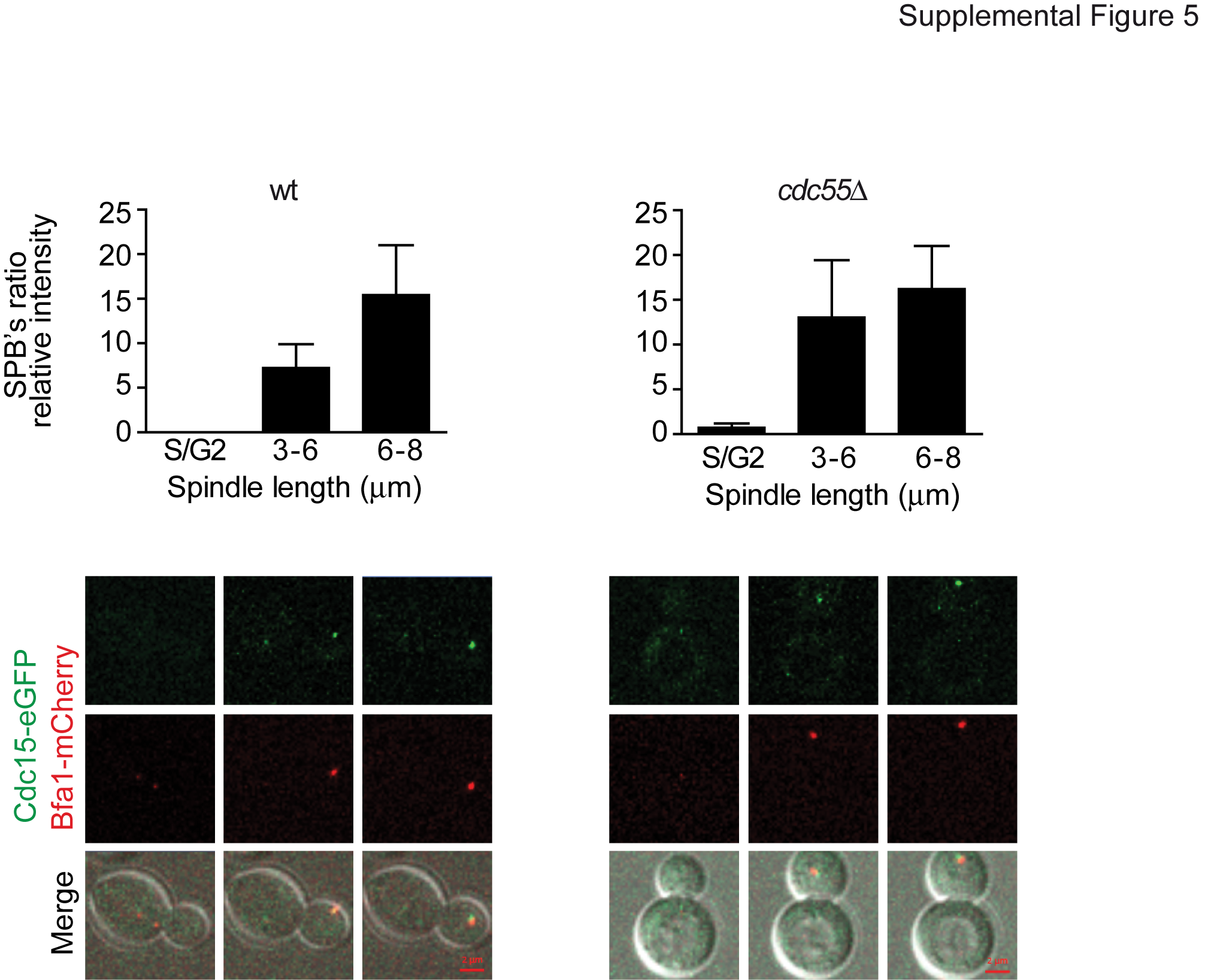

Supplement: Figure S5 — Increased Cdc15 asymmetric localization in the absence of Cdc55 in a synchronous cell cycle after G1 release. Strains Y911 (MATa CDC28F19 CDC14-myc9 CDC15-eGFP BFA1-mCherry) and Y957 (as Y911, but cdc55Δ) were arrested at G1 with α-factor and released into a synchronous cell cycle. Time-lapse microscopy was performed as described in the Material and Methods. (TIF) [file pgen.1003966.s005.tif]
